# Differential expression of viral entry protein neuropilin 1 (NRP1) and neuropilin 2 (NRP2) in fatal COVID-19

**DOI:** 10.1128/jvi.01384-25

**Published:** 2025-10-29

**Authors:** A. Dette, F. Moers, T. Mayr, S. v. Stillfried, M. Bernhardt, S. Förster, C. Werlein, M. Ackermann, M. H. Muders, G. Kristiansen, P. Boor, I. Gütgemann

**Affiliations:** 1 Institute of Pathology, University Hospital Bonn 197696, Bonn, Germany; 2 Institute of Pathology, RWTH Aachen University Hospital 610384, Aachen, Germany; 3 Institute of Pathology, Hannover Medical School 216543, Hanover, Germany; 4 Institute of Pathology and Department of Molecular Pathology, Helios University Clinic Wuppertal 60865, Wuppertal, Germany; 5 Asklepios Medical Care Center Pathology, Gauting, Germany; 6 Precipoint Innovation GmbH, Garching, Germany; St Jude Children's Research Hospital, Memphis, Tennessee, USA

**Keywords:** COVID-19, PASC, neuropilins, NRP1, NRP2, SARS-CoV-2, spike protein, CODEX, scRNA

## Abstract

Coronavirus disease 2019 (COVID-19) is associated with hyperinflammation, endothelialitis, hypoxemia, and hypercoagulation, contributing to thrombosis in acute severe and long COVID. While ACE2 is the primary severe acute respiratory syndrome coronavirus 2 (SARS-CoV-2) receptor, its low expression in certain infected cell types suggests alternative co-receptors. Neuropilins (NRP1 and NRP2), widely expressed, have been proposed as co-factors for viral entry. We analyzed NRP1 and NRP2 expression in autopsy tissues from heart, lung, and hematolymphoid organs using immunohistochemistry (*n* = 20) and compared findings with public single-cell RNA sequencing (scRNAseq) data. Selected cases were further examined by spatial multiplex immunofluorescence (CODEX). *In vitro* binding of NRP1/NRP2 to SARS-CoV-2 spike fragments S1 and S1′ was assessed by immunofluorescence microscopy. NRP1 was abundantly expressed in myocardial capillary endothelial cells (ECs) and macrophages in the heart and lung; NRP2 was found in alveolar macrophages and mast cells. scRNAseq re-analysis confirmed these *in situ* patterns. *In vitro*, NRP1 exclusively bound S1, while NRP2 bound both S1 and S1′. SARS-CoV-2 RNA was detected in neuropilin-positive, *ACE2*/*TMPRSS2*-negative vascular EC and mast cells. The detection of SARS-CoV-2 RNA in neuropilin-positive but *ACE2*/*TMPRSS2*-negative cell clusters supports that neuropilins are involved in systemic viral dissemination. NRP1 on vascular EC may contribute to angiogenesis, vascular damage, and microangiopathy, while NRP2 represents a potential immunomodulatory target to regulate macrophage activity, resolve inflammation, and potentially prevent the progression of pulmonary fibrosis and limit excessive mast cell activation in long COVID.

**IMPORTANCE:**

The well-known severe acute respiratory syndrome coronavirus 2 (SARS-CoV-2) receptor, angiotensin-converting enzyme 2 (ACE2), exhibits low expression in key cell types implicated in coronavirus disease 2019 (COVID-19) pathology, such as endothelial cells and B cells, macrophages, and mast cells. In contrast, neuropilins, identified as co-receptors for SARS-CoV-2, are abundantly expressed in these cells under physiological conditions and may be involved in virus-host interactions. This study presents a detailed *in situ* analysis of Neuropilin 1 (NRP1) and Neuropilin 2 (NRP2) expression in fatal COVID-19 cases using immunohistochemistry and spatial multiplex immunofluorescence phenotyping, complemented by single cell RNA sequencing. Additionally, it demonstrates differential binding affinities of NRP1 and NRP2 to SARS-CoV-2 spike protein fragments S1 and S1′ *in vitro*, suggesting distinct roles for these neuropilins in viral recognition. This study highlights the impact of the unique furin cleavage site in SARS-CoV-2, which may contribute to increased pathogenicity through its interaction with NRP1.

## INTRODUCTION

Severe acute respiratory syndrome coronavirus 2 (SARS-CoV-2) caused the coronavirus disease 2019 (COVID-19) pandemic and emerging variants remain a persistent health threat. SARS-CoV-2 infection triggers a cascade of pathophysiological mechanisms, including endothelial dysfunction, hyperinflammation, and platelet activation, which collectively contribute to immunothrombosis (1). Although SARS-CoV-1 and SARS-CoV-2 genome sequences are highly homologous, SARS-CoV-2 exhibits a 10-20-fold higher binding affinity to the angiotensin-converting enzyme 2 (ACE2) receptor compared with SARS-CoV-1 due to differences in its spike protein. This enhances cell entry and results in a more vigorous pro-inflammatory cytokine response (“cytokine storm”) in monocytes and macrophages, accompanied by a dysregulated myeloid cell compartment (2–4). Monocytes also play a central role in inducing COVID-19-associated microvascular thrombosis by initiating intrinsic and extrinsic coagulation cascades (5). While fatal COVID-19 cases have declined, post-acute sequelae of COVID-19 (PASC) (6) were estimated to affect at least 10% of infected people—65 million individuals globally, including symptoms related to microvascular damage, e.g. dyspnea, “brain fog,” and thrombotic complications (1, 7). Survivors of severe COVID-19 often experience variable degrees of health impairment, with pulmonary fibrosis as a significant long-term complication (8).

SARS-CoV-2 typically enters cells via its spike protein binding to the ACE2 receptor, with subsequent processing by transmembrane protease serine subtype 2 (TMPRSS2; Fig. S1) (9). While *ACE2* expression has been extensively studied (10, 11), the protein is absent in several cell types affected by the virus, including neurons (12) and nasal and respiratory epithelia (13), and is low or absent in capillary endothelial cells (ECs) (13, 14). Neuropilin-1 (NRP1) has been identified as a co-factor that facilitates SARS-CoV-2 entry via furin-processed S1 spike protein fragments (13, 15). Following cleavage at the S1/2 site (16), a C-terminal sequence (-RRAR*) is exposed, conforming to the C-end rule (CendR) motif (-RXXR*) (13, 15, 17). Binding of the S1 fragment to the b1/b2 domains of NRP1 and NRP2 has been shown to enhance viral infectivity and replication (15, 18). While several viruses use neuropilins for cell entry, only Epstein-Barr virus (EBV) and human T-lymphotropic virus 1 (HTLV1) are known to interact with neuropilins via the CendR motif (19).

Neuropilins are transmembrane proteins that lack intrinsic signaling activity and function as co-receptors for signaling molecules like vascular endothelial growth factors (VEGF) and class 3 semaphorins. They are involved in angiogenesis, lymphangiogenesis, immune responses, axon guidance (20, 21), as well as cancer progression and therapy resistance (22). Organ-specific SARS-CoV-2 pathogenesis depends on the expression of viral entry factors. NRP1 is predominantly expressed in vascular ECs, vascular smooth muscle cells, mesenchymal stem cells, neurons, epithelial cells of the respiratory and gastrointestinal tracts (23), as well as pancreatic β cells (24). In addition to its neuronal expression (25), NRP2 is expressed in smooth muscle cells of the intestine and bladder (26) and is essential for the development of smaller lymphatic vessels (20). The absence or low levels of *ACE2* expression and presence of neuropilins on respiratory and olfactory epithelia and vascular ECs (10, 13, 14), combined with the upregulation of neuropilin transcripts in SARS-CoV-2 infected lungs (27), highlight the critical role of neuropilins in systemic and acute COVID-19. Despite organ-specific studies on ACE2 and other SARS-CoV-2 co-factors (28), detailed characterization of NRP1 and NRP2 expression in COVID-19-infected tissues remains limited.

This study aims to provide a comprehensive assessment of NRP1 and NRP2 protein expression in the heart, lung, and hematolymphoid organs of COVID-19 autopsies using immunohistochemistry (IHC). Selected cases also underwent *in situ* multiplex immunofluorescence staining (CODEX) (29) to visualize rare cell types at a spatial level. The findings were compared with single-cell RNA sequencing (scRNAseq) data from fatal COVID-19 heart and lung samples (30). In addition, SARS-CoV-2 viral RNA was detected and localized in lymph nodes and spleen using RNAscope *in situ* hybridization. Finally, the role of neuropilins in viral recognition was further explored *in vitro* by analyzing their differential binding to spike protein fragments using immunofluorescence microscopy.

## RESULTS

### Histopathologic changes in COVID-19 autopsies with a focus on neuropilins

Pulmonary tissue from fatal COVID-19 autopsies revealed alveolar damage in fulminant cases, as well as early-phase organizing chronic pneumonia in subacute cases. Histopathological features included prominent intra-alveolar edema, microthrombi, hyaline membranes, intra-alveolar fibrin deposits, and mixed inflammatory infiltrates. The myocardium demonstrated scattered mixed inflammatory cells alongside either normal or hypertrophic myocardial fibers. Similar histomorphological findings have been described previously (27, 31, 32).

To investigate NRP1 and NRP2 expression patterns *in situ*, standard IHC was performed on COVID-19 autopsy tissues, as summarized in Table 1. Neuropilins were expressed on epithelial, mesenchymal stromal, and immune cells in all organs examined (Table 1) with some notable differences: NRP1 was strongly expressed in macrophages and Kupffer cells, splenic white pulp and lymph node follicular lymphocytes, capillary and liver sinusoidal ECs, as well as in proximal tubules and glomeruli (Fig. S5). In contrast, NRP2 expression was more restricted and was moderately detected in intestinal smooth muscle cells and in macrophages within the lung, trachea, spleen, lymph nodes, and adrenal gland (Fig. S6).

**TABLE 1:** Overview of NRP1 and NRP2 expression *in situ* in fatal COVID-19^*a*^

Organ	NRP1	Figure	NRP2	Figure
Heart	ECs, mononuclear cells	1A, S5B, 1B, 1D	Few mononuclear cells	S6B
Lung	Alveolar macrophages, interstitial macrophages, ECs heterogeneously	2B, S5A	Alveolar macrophages, interstitial macrophages, smooth muscle of bronchi, perineurium, few ECs, smooth muscle of vessels	2C, S6A
Kidney	Epithelium of proximal tubule, glomerular cells	S5C	Few cells in glomerulus	S6C
Liver	Macrophages, sinusoidal endothelium	S5D	No staining	S6D
Spleen	Lymphocytes predominantly in white pulp, macrophages	S5E	Sparse scattered mononuclear cells	S6E
Lymph node	Lymphocytes in lymph follicles, sinus histiocytes, macrophages, ECs	S5F	Sinus histiocytes, lymphatic ECs	S6F
Colon	ECs in submucosa, mononuclear cells in lamina propria	S5G	Smooth muscle of muscularis propria, mononuclear cells in lamina propria	S6G
Adrenal gland	ECs, macrophages	S5H	Cortical epithelium, macrophages	S6H
Trachea	ECs in submucosa, sparse cells in respiratory epithelium, mononuclear cells in submucosa and in epithelium	S5I	Respiratory epithelium, few ECs, mononuclear cells in submucosa	S6I

^*a*^ Identification of tissue and cell type was based on histopathological and morphological evaluation of tissues from patients 1–3 (Table S1A) by two independent pathologists. Representative images of corresponding morphological structures are listed.

### Increased NRP1 expression in capillaries of fatal COVID-19 hearts

**Fig 1:**
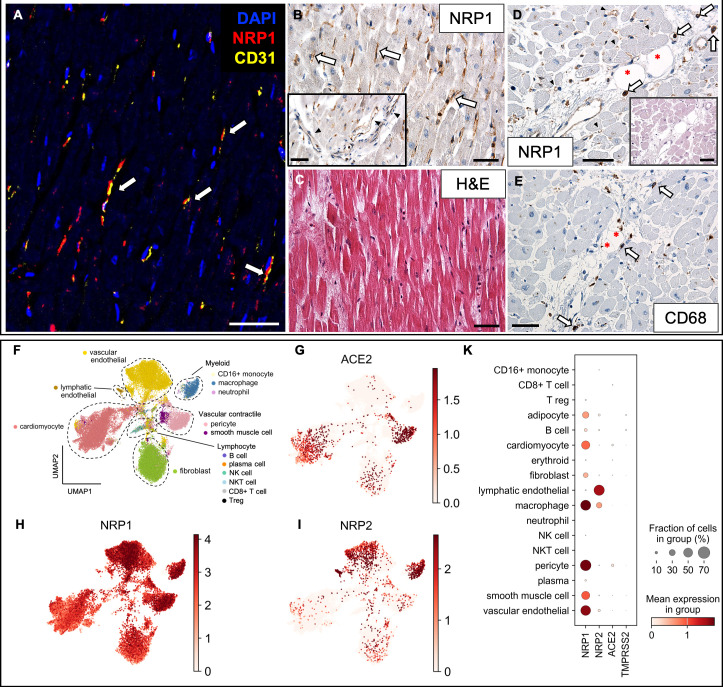
NRP1 is expressed on endothelial and mononuclear cells in fatal COVID-19 cardiac autopsy tissue. (**A**) CODEX image with DAPI displayed in blue, NRP1 in red and CD31 in yellow. Co-localization of NRP1 and the endothelial marker CD31/PECAM1 (indicated by arrows). (**B**) Capillaries (arrows) and larger vessels (inset; arrowheads) expressed NRP1 by IHC. (**C**) H&E staining corresponding to B. (**D**) NRP1 expression in larger vessels and small capillaries (arrowheads) and mononuclear cells (arrows). Corresponding H&E stain in inset. (**E**) CD68-positive macrophages lining the vascular bed of a larger vessel (arrows). Adipocytes are marked by asterisks (*). **A–C**, patient 2; **D–E**, patient 12 (Table S1A). Magnification 400× for **B–E**. Scale bars correspond to 50 µm. (**F**) Automatic cell classification identified 19-cell subsets across compartments in re-analyzed data set of fatal COVID-19 hearts (30). No annotated cluster for adipocytes and erythroid cells. Gene expression along clusters for *ACE2* (**G**), *NRP1* (**H**), and *NRP2* (**I**). (**K**) Dot plot summarizing the expression levels with dot size indicating the percentage of cells expressing the relevant gene and saturation corresponding to average expression level. All plots were generated using the Scanpy package in Python.

Myocardial small capillaries, arterioles, and postcapillary venous vessels showed strong NRP1 expression by IHC in COVID-19 (Fig. 1B), whereas NRP2 was not detected (Fig. S6B). NRP1 localization on vascular EC was confirmed by CD31 co-expression using CODEX multiplex imaging (Fig. 1A). scRNAseq analysis demonstrated abundant *NRP1* transcripts in vascular EC and pericytes, with *NRP2* mainly restricted to lymphatic EC (Fig. 1H through K). Although *NRP1* mRNA was expressed in cardiomyocytes, neither NRP1 nor NRP2 protein was detected by IHC or CODEX. In the myocardium, NRP1-positive mononuclear cells along larger vascular beds (Fig. 1D) corresponded to CD68-positive macrophages in serial sections (Fig. 1E). scRNAseq analysis confirmed strong expression of *NRP1* and *NRP2*—but not *ACE2*—in cardiac macrophages (Fig. 1G through K). *ACE2* transcripts were largely confined to cardiomyocytes and pericytes, consistent with prior findings (33), and barely detectable in vascular EC (Fig. 1G). Compared with influenza pneumonitis and non-infected control myocardium, NRP1-positive capillaries were more frequent in COVID-19 (*F* (2, 23) = 0.91, *P* = 0.417); Fig. S7).

### Expression patterns suggest an important role for neuropilins in macrophages in fatal COVID-19 lungs

**Fig 2:**
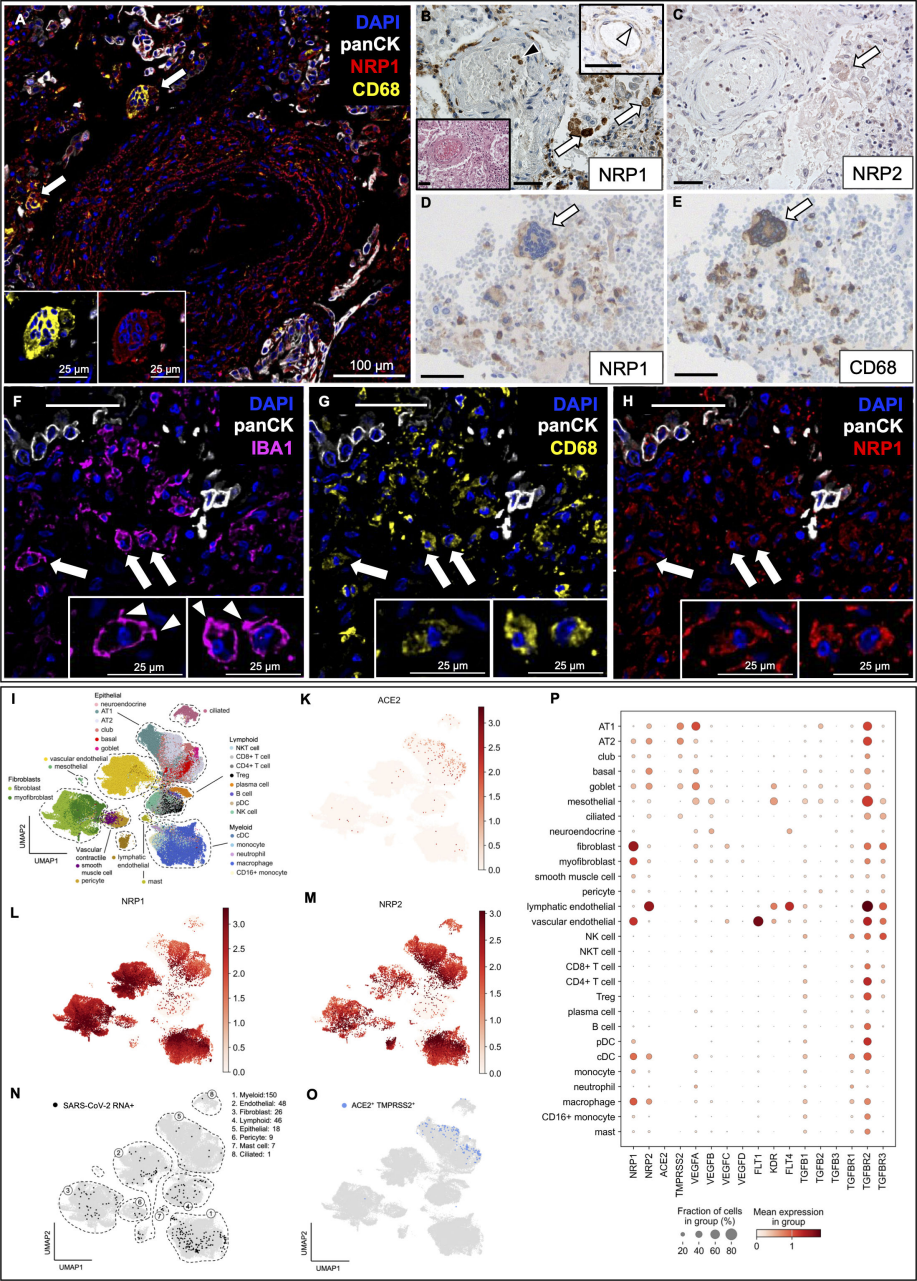
Alveolar and syncytial macrophages express NRP1 and NRP2 in fatal COVID-19 lung autopsy tissue. (**A**) CODEX image showing syncytial macrophages co-expressing CD68 and NRP1 (arrows, insets). (**B, C**) IHC of the same lung vessel with corresponding H&E inset. (**B**) NRP1 protein in alveolar macrophages (arrows) and blood mononuclear cells (black arrowhead). Upper inset: NRP1 expression in vascular endothelium (white arrowhead). Lower inset: H&E stain of corresponding region. (**C**) IHC of NRP2 expression in a syncytial macrophage (arrow) in serial section of B. IHC of NRP1 (**D**) and CD68 (**E**) positive syncytial macrophage in the lung (arrows). (**F–H**) CODEX images highlighting macrophages in the lung with co-expression of IBA1 (**F**), CD68 (**G**), and NRP1 (**H**). Arrowheads indicate macrophages with ruffled membrane segments. **A–C** and **F–H**, patient 3; **D–E**, patient 20 (Table S1A). Scale bars correspond to 50 µm unless stated otherwise. CODEX color code: nuclei (DAPI, blue), pancytokeratin (panCK, white), NRP1 (red), IBA1 (magenta), CD68 (yellow). (**I**) Automatic cell classification identified 28-cell subsets across compartments in re-analyzed data set of fatal COVID-19 lungs (30). Gene expression along clusters for *ACE2* (**K**), *NRP1* (**L**), and *NRP2* (**M**). (**N**) UMAP embedding showing SARS-CoV-2 RNA^+^ cells (black dots). (**O**) UMAP plot showing co-expression of acknowledged SARS-CoV-2 entry factors *ACE2* and *TMPRSS2*. SARS-CoV-2 RNA (**N**) was detected in cells devoid of *ACE2* and *TMPRSS2* expression. (**P**) Dot plot summarizing expression of genes associated with SARS-CoV-2 and neuropilin pathways. *FLT1*, VEGF receptor 1; *KDR*, VEGF receptor 2; *FLT4*, VEGF receptor 3. All plots were generated using the scanpy package in Python.

NRP1 and NRP2 proteins were heterogeneously expressed in larger vessel EC of the trachea and lung of COVID-19 patients (Fig. 2B inset; Fig. S5I and S6I), while absent from small capillaries. Their transcripts were detected in vascular EC (Fig. 2L and M). Intense NRP1 expression in alveolar macrophages was not specific to COVID-19 but was also noted in other causes of death (*n* = 28, Fig. S2). However, we identified COVID-19-associated syncytial macrophages with high phagocytic activity in the lung that were positive for NRP1 (Fig. 2A inset, 2D) and CD68 (Fig. 2A inset, 2E) by CODEX and IHC. NRP1/CD68-positive macrophages, predominantly interstitial, expressed the microglial and macrophage marker ionized calcium-binding adapter molecule 1 (IBA1) in their cytoplasm, with ruffled membrane appearance (Fig. 2F through H), suggestive of migratory behavior (34).

NRP2 protein expression was also detected in alveolar macrophages (Fig. 2C; Fig. S6A), consistent with scRNA-seq analysis (Fig. 2M and P). *NRP2* transcripts were mainly expressed in lymphatic ECs (Fig. 2P), while *NRP1* mRNA was predominantly expressed by vascular ECs, fibroblasts, and myofibroblasts (Fig. 2P). Alveolar type II (AT2) and goblet cells also expressed *NRP1* mRNA (Fig. 2P), although NRP1 protein was largely absent in lung epithelial cells (Fig. S5A). Focal, weak NRP2 protein expression in epithelial cells, detected by IHC, was confirmed by transcript data (Fig. 2P).

*ACE2* and *TMPRSS2* mRNA were largely restricted to lung epithelial cells (Fig. 2K, O and P), while ECs, stromal cells, as well as lymphocytes and macrophage populations lacked expression. No overlap was observed between SARS-CoV-2-infected cell types (Fig. 2N) and those co-expressing *ACE2* and *TMPRSS2* (Fig. 2O). Only low levels of SARS-CoV-2 RNA were detected in lung epithelial cells from fatal COVID-19 patients (Fig. 2N).

We detected a significant upregulation of *NRP1* transcripts in vascular ECs in COVID-19 lungs compared with noninfectious controls (*P* < 7.5E-6, Fig. S8A), while *ACE2* and *TMPRSS2* expression remained unchanged. *NRP1* and the vascular endothelial marker *FLT1*/*VEGFR1* (fms-related receptor tyrosine kinase 1/VEGF receptor 1) transcript expression predominated in vascular ECs, while lymphatic ECs showed high *NRP2* expression along with the lymphatic markers *KDR*/*VEGFR2* (kinase insert domain receptor/VEGF receptor 2) and *FLT4*/*VEGFR3* (fms related receptor tyrosine kinase 4/VEGF receptor 3) (Fig. 2P). The activating ligand for VEGFRs, *VEGFA*, was produced and upregulated (*P* < 7.5E-6) by lung epithelial cells (Fig. 2P).

Both vascular and lymphatic ECs expressed high levels of the transforming growth factor beta receptor 2 (*TGFBR2)* (Fig. 2P), suggesting endothelial repair in response to viral infection (35). *TGFBR2* was significantly upregulated in vascular ECs (*P* < 7.5E-6, Fig. S8A) in fatal COVID-19, while upregulation of the neuropilin-binding ligand *TGFB1* (20) was observed in monocytes (Fig. S8B). Monocytes also showed upregulation of the *TGFB1* inducing factor C-C motif chemokine ligand 2 (*CCL2)* (*P* < 7.5E-6, Fig. S8B) (36).

### SARS-CoV-2 RNA and NRP1 expression in lymphocytes in hematolymphoid organs

**Fig 3:**
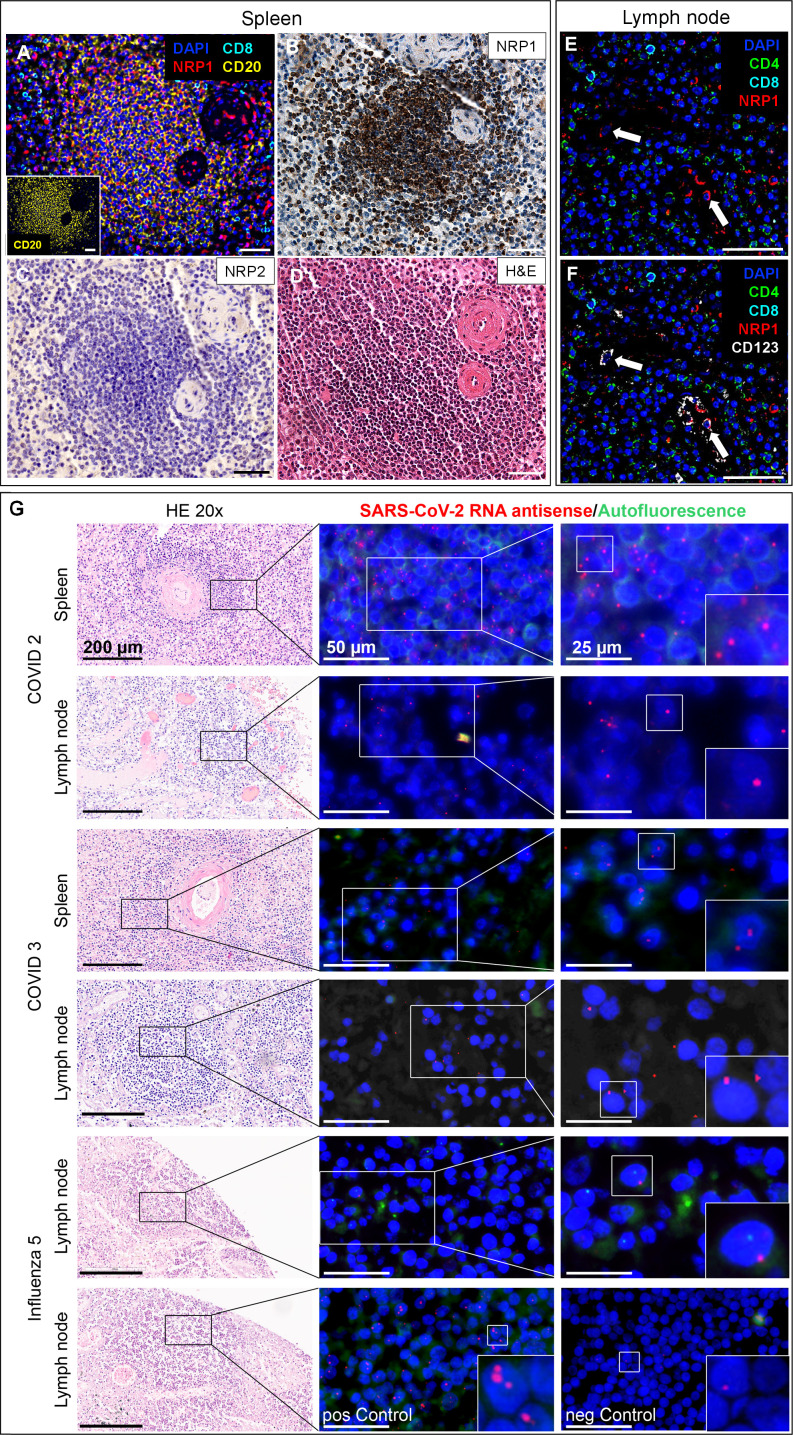
NRP1 expression on lymphocytes may enable SARS-CoV-2 infection in the spleen and lymph node of fatal COVID-19. (**A**) CODEX image of a lymph follicle in the spleen in fatal COVID-19. A subset of B lymphocytes co-expressed NRP1, while cytotoxic T cells were NRP1 negative. The inset shows that the depicted lymph follicle is largely composed of CD20-positive B cells. (**B**) Corresponding region in NRP1 IHC revealed strong NRP1 expression in follicular lymphocytes, while the same region was negative for NRP2 (**C**). (**D**) H&E stain of corresponding serial section. (**E**) CODEX image of a lymph node from a fatal COVID-19 case. CD8 and CD4 T cells did not co-express NRP1, while pDCs in lymph node, identified by the expression of CD123, co-expressed NRP1 (**F**). Scale bars correspond to 50 µm unless stated otherwise. **A–D**, COVID-19 patient 2; **E–F**, COVID-19 patient 3 (Table S1A). CODEX color code: nuclei (DAPI, blue), NRP1 (red), B lymphocyte marker CD20 (yellow), cytotoxic T-cell marker CD8 (cyan), helper T-cell marker CD4 (green) and CD123 (white). (**G**) RNA scope analysis of spleen and lymph node from fatal COVID-19 cases (patient 2 and 3; Table S1A) and influenza-related death (patient 5; Table S1B) with corresponding areas in H&E (left column), using serial sections. In COVID-19 cases, lymphocytes within the spleen and lymph nodes displayed positive RNAscope signals for SARS-CoV-2 RNA. In contrast, lymph node tissue from the influenza patient was largely negative. Bottom row: RNAscope probes with defined targets were applied to influenza lymph node tissue (patient 5), including a positive control probe set (3-plex targeting *POLR2A*, *PPIB*, and *UBC*) and a negative control probe (targeting the *dapB* gene of *Bacillus subtilis*). Scale bars are provided per column. FISH images in the far-right column show a twofold magnification of the middle column with areas of interest highlighted in white boxes.

Similar to the lung, macrophages and sinus histiocytes in the lymph nodes of COVID-19 autopsies were strongly positive for NRP1 (Fig. S5F) and NRP2 (Fig. S6F). Follicular lymphocytes in lymph nodes and spleen were NRP1-positive and NRP2-negative (Fig. 3B and C; Fig. S5E). NRP1 expression in lymphocytes was not specific to COVID-19 but was also observed in influenza and noninfectious causes of death. CODEX multiplex analysis revealed that a subset of CD20-positive splenic B lymphocytes co-expressed NRP1 (Fig. 3A), while CD8- and CD4-positive lymphocytes were negative for NRP1 (Fig. 3E). Lymphocytes in the spleen and lymph nodes showed antisense SARS-CoV-2 RNA signals, as demonstrated by RNAscope FISH (Fig. 3G).

### NRP1 and NRP2 expression in rare cell types linked to COVID-19 phenotype

**Fig 4:**
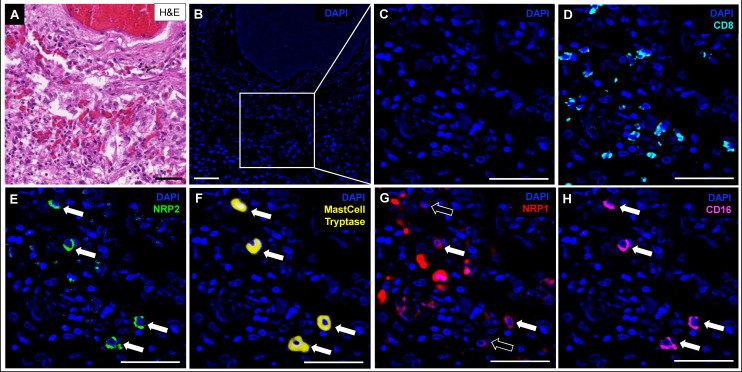
Mast cells strongly express NRP2. (**A**) H&E staining of lung tissue from a fatal COVID-19 case. (**B**, **C**) Corresponding region in CODEX. (**D**) CD8+ T-cell infiltrate in surrounding area. (**E**) NRP2-positive cells (arrows). (**F**) Mast cell tryptase identified NRP2-positive cells as mast cells. (**G**) A subset of mast cells also expressed NRP1 (white arrows). NRP1-negative mast cells are marked with black arrows. (**H**) Mast cells were also positive for CD16. **A–H**, patient 2 (Table S1A). Scale bars correspond to 50 µm. CODEX color code: DAPI (blue), CD8 (cyan), NRP2 (green), mast cell tryptase (yellow), NRP1 (red), and CD16 (magenta).

Severity of COVID-19 inversely correlates with the plasmacytoid dendritic cell (pDC) response (37). Tissue DCs in autopsy samples, identified by CD123 expression by CODEX (Fig. 3F), were positive for NRP1 in lymph nodes (Fig. 3E) but were NRP2-negative. NRP1 expression was also detected in tracheal DCs by IHC (Fig. S5I). Transcript analysis revealed *NRP1* but not *NRP2* expression in pDCs (Fig. 2P). Although *NRP1* transcripts were present in mast cells (Fig. 2L and P), protein expression was not detected in a subset of mast cells that instead exhibited strong NRP2 expression by CODEX (Fig. 4E through G).

### NRP1 and NRP2 differentially bind to SARS-CoV-2 cleavage sites S1 and S1′

**Fig 5:**
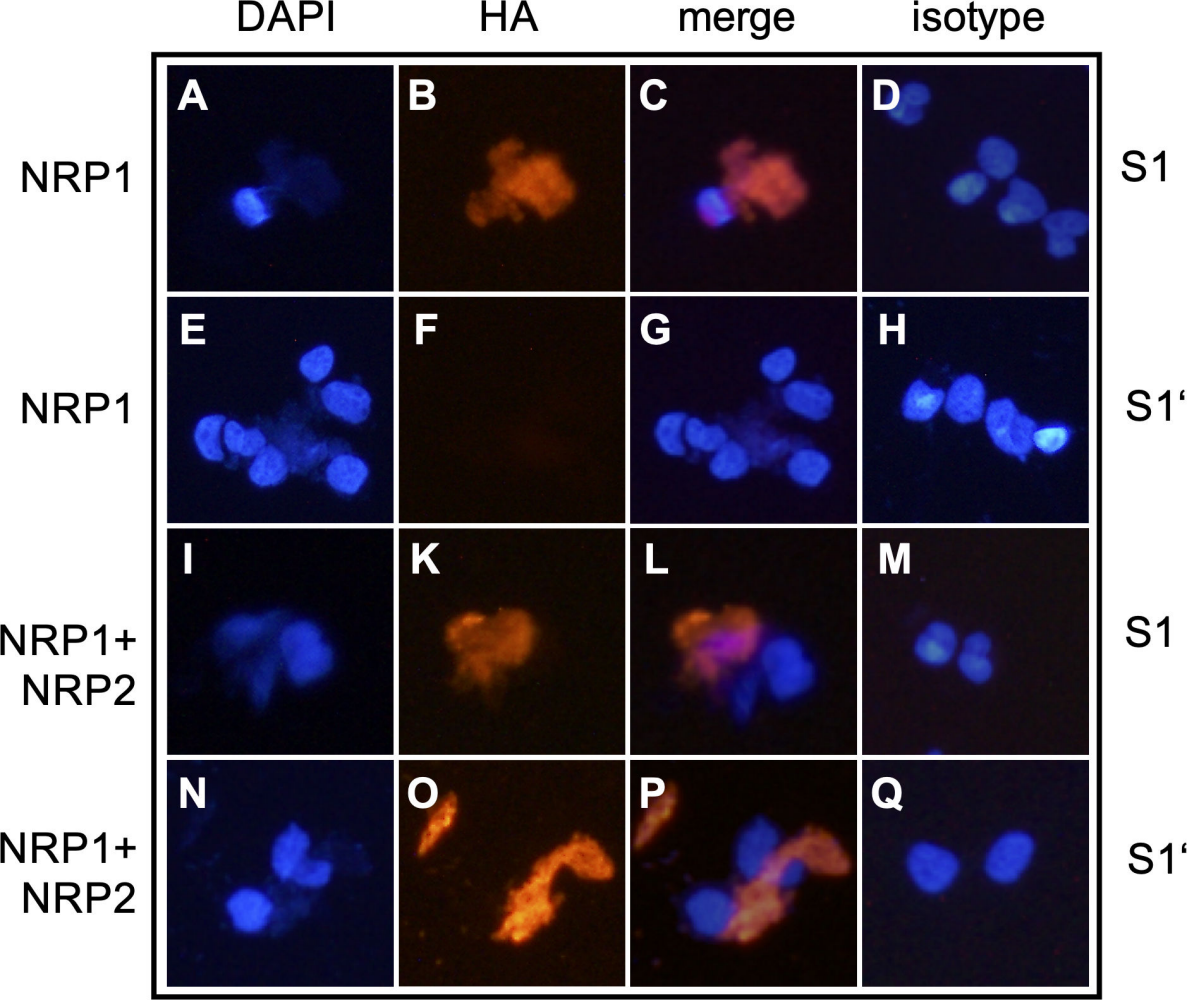
Binding of soluble S1′ spike fragment requires presence of NRP2. HEK293 cells endogenously expressing NRP1 (Fig. S3 and S4) bound soluble S1 spike protein (**A–C**) but not soluble S1′ (**E–G**). HEK293 cells stably transfected to express NRP2 (Fig. S3 and S4) bound soluble S1 (**I–L**) and also S1′ (**N–P**). DAPI nuclear staining (**A, E, I, N**). Anti-HA IF for S1 or S1′ (**B, F, K, O**); merged DAPI/IF (**C, G, L, P**); isotype controls (**D, H, M, Q**).

To explore the role of soluble S1 and S1′ in neuropilin binding (Fig. S1), NRP2-negative HEK293 cells, which endogenously express NRP1, were transfected to express NRP2. These cells express low to undetectable levels of the SARS-CoV-2 (co-)receptors *ACE2*, *FURIN,* and *TMPRSS2* (Fig. S3). HEK293 cells not expressing NRP2 (Fig. S4I) served as internal negative controls to quantify NRP2 binding to spike fragments. As previously shown (13, 15), endogenous NRP1 on HEK293 cells bound the soluble S1 spike fragment (Fig. 5A through C). However, S1′ was not bound by NRP1 (Fig. 5E through G). Only recombinant expression of NRP2 enabled HEK293 cells to additionally bind S1′ soluble spike fragment (Fig. 5N through P), with preserved S1 binding (Fig. 5I through L). Manual cell counts revealed NRP2 expression and S1′ binding in a similar fraction of NRP2-transfected cells (5%; 4%).

## DISCUSSION

This study presents a comprehensive analysis of NRP1 and NRP2 protein expression in COVID-19 *in situ* by IHC and CODEX, alongside transcriptomic data from publicly available scRNAseq data sets (30). Our findings highlight the critical role of neuropilins in SARS-CoV-2 infection within the lung, heart, and hematolymphoid tissues. We observed concordant NRP1 and/or NRP2 protein and transcript expression, especially in cell types with minimal *ACE2* expression, including vascular ECs, macrophages, B lymphocytes, and mast cells.

Primary targets of SARS-CoV-2 infection are nasopharyngeal, bronchial, and lung epithelial cells, with ACE2 and TMPRSS2 as the primary entry factors (Fig. 2O) (9). However, minimal *ACE2* and *TMPRSS2* expression in SARS-CoV-2-positive cell clusters (Fig. 2N and O) suggests neuropilins, among others, as alternative entry factors. It should be noted that productive infection of epithelial cells can induce severe cytopathic effects, potentially hindering their detection in scRNA-seq analyzes. Our findings demonstrate the presence of SARS-CoV-2 RNA within vascular ECs, macrophage, B lymphocyte, and mast cell clusters expressing neuropilins, further supporting their role in viral entry (Fig. 2L and M). While monocytes and lymphocytes are reported to lack *ACE2* expression (38), consistent with our own findings (Fig. 2K and P), we observed strong neuropilin expression in B lymphocytes and macrophages (Fig. 1 to 3). We detected antisense SARS-CoV-2 RNA in lymphocytes in spleen and lymph nodes. While the precise identity and localization of the signal remain uncertain, B lymphocytes are known to be permissive to productive SARS-CoV-2 infection (39), and NRP1 expression provides a potential mechanism for the severe lymphopenia associated with poor outcomes in COVID-19 patients (40). Nrp1 is also a surface marker of CD4+ CD25+ T regulatory cells (Tregs) and is co-regulated with *Foxp3* (forkhead box P3), an important regulator for Tregs (41). The potential role of Tregs in severe COVID-19 and PASC has been discussed recently (42), including findings on T-cell dysregulation in PASC (43). However, contrary to previous results (41), we did not detect relevant *NRP1* mRNA in Tregs in COVID-19 lungs (Fig. 2P).

The importance of alternative docking receptors aligns with previous reports of SARS-CoV-2 virus particles in vascular ECs despite low ACE2 levels (27). Biochemical studies (15) indicate that NRP1 enhances infectivity in the presence of ACE2 by binding the S1 peptide to its b1 domain but does not facilitate viral attachment independently. These results were later extended, showing that NRP1 interacts with the receptor binding domain (RBD) and full trimeric spike protein with higher binding frequency than ACE2, thus enhancing viral attachment *in vitro* (44). Similarly, NRP2 has been shown to interact with the RBD of SARS-CoV-2 spike protein *in vitro* (45), indicating that neuropilins may serve roles beyond simple co-factors. Our *in vitro* studies (Fig. 5) confirm the binding of NRP1 to the novel and SARS-CoV-2-specific S1 spike fragment (Fig. S1A), whose C-terminus (-PRRAR*; ProArgArgAlaArg*) complies with the CendR consensus sequence (-RXXR*) (17). Proteolytic cleavage at the S1′/S2′ site generates a non-canonical CendR motif at the C terminus of S1′ (-KPSKR*; LysProSerLysArg*), which is conserved in SARS-CoV-1 (16) and is only bound in the presence of NRP2 (Fig. 5). NRP1 lacked binding capacity for S1′. This suggests greater flexibility of NRP2 towards CendR-like peptides, consistent with its binding to the C-terminal end of mature VEGFC protein (46), which does not conform to the CendR consensus (17). The emergence of the S1/S2 cleavage site in SARS-CoV-2 resulted in S1 peptide and *de novo* NRP1 binding. After deletion of the S1/S2 cleavage site, there is still evidence of S2′ spike proteolytic fragments, which suggests the existence of S1′ *in vitro* (47). Additionally, Frolova et al. (2022) showed that after deletion of the S1/S2 cleavage site, syncytia formation is still observed in cells overexpressing TMPRSS2. This study also reported the presence of a band reminiscent of S2′, suggesting that cleavage at S1/S2 (furin cleavage site) is not an absolute requirement for cleavage at S1′/S2′ (48). Our data indicate that, apart from a differential expression of neuropilins throughout the body, differential recognition motifs for viral peptides may also contribute to COVID-19 pathology. The ability of S1′ to bind via NRP2, but not NRP1 alone, suggests differential interactions between the two neuropilins and CendR-compatible proteins. This may also apply to other viruses exploiting neuropilin-mediated cellular uptake (19).

More abundant NRP1 expression *in situ* compared with NRP2 (Fig. 1 and 2; Table 1; Fig. S5 and S6) may reflect differences relevant to clinical severity and viral dissemination. It is possible that not only the cell tropism based on NRP1 and NRP2 expression contributes to the pathology of acute COVID-19 and PASC, but also the differential binding of neuropilins to proteolytic spike fragments. Future studies should address whether soluble circulating spike proteins, observed in PASC patients (6), interact with neuropilins on ECs, potentially contributing to chronic microangiopathy.

Although neuropilin expression patterns were similar in COVID and non-COVID-associated death, we identified a trend toward increased NRP1 protein levels in myocardial capillary ECs (Fig. S7) and transcriptional upregulation in pulmonary vascular ECs in COVID-19 (Fig. S8A), consistent with prior findings (27). High NRP1 expression in cardiac microcapillaries (Fig. 1B, H and K), combined with scarce *ACE2* expression (Fig. 1G and K), supports earlier reports of low or negligible *ACE2* transcripts in ECs (10, 13, 14, 33). Occasional detection of ACE2 protein despite minimal transcript detection (49, 50) may reflect potential methodological challenges due to different antibodies.

SARS-CoV-2 infection triggers a cytokine storm, complement over-activation, and thrombosis, leading to endothelial damage with tissue hypoxia, angiogenesis (51, 52), and vascular remodeling (27, 32). Werlein et al. (32) reported intussusceptive angiogenesis in COVID-19 hearts alongside increased CD11b/TIE2 positive macrophages near vessels, suggesting a promoting role in driving angiogenesis. Interestingly, we observed a subset of cardiac macrophages strongly positive for NRP1 in the vicinity of larger vessels (Fig. 1D). Also in the lung, intussusceptive angiogenesis in COVID-19 has been demonstrated, accompanied by an upregulation of *NRP1*, *NRP2,* and *VEGFA* (27), in partial agreement with our own findings of NRP1 and NRP2 protein and transcript expression in the lung (Fig. 2; Fig. S8A). Autopsy results revealed small vessel occlusion and microangiopathy alongside endothelial damage to be involved in the pathogenesis of COVID-19 (5). Endothelial Nrp1 is essential for sprouting angiogenesis (53). Even without replicating virus, spike protein binding to neuropilins may initiate signaling. The interplay between virus-host interactions and NRP1 expression may contribute to initial micro-thrombotic events, intussusceptive angiogenesis, acute and chronic hypoxemia, and subsequent tissue remodeling and fibrosis (54), linking acute infection to long-term sequelae such as PASC.

Another observed complication of acute COVID-19 with long-term consequences is new-onset hyperglycemia and diabetes. Wu et al. (2021) (24) found that pancreatic β cells exhibit selectively high expression of NRP1, with low ACE2 and TMPRSS2 expression at both mRNA and protein levels. They also showed that SARS-CoV-2 specifically infects pancreatic β cells and induces apoptotic cell signaling, which was reduced by NRP1 inhibition, supporting NRP1-mediated β cell targeting.

NRP2 is upregulated in macrophages and during pro-inflammatory states in the heart and lung (55). Therapeutic modulation of NRP2 has been discussed for myelofibrosis (56) and has shown promise in late-stage clinical trials for pulmonary fibrosis in sarcoidosis patients (55), where the possible mechanism of action of the NRP2 agent Efzofitimod is to reduce pro-inflammatory macrophages and to prevent the progression of pulmonary fibrosis (55). We observed abundant NRP1 and NRP2 expression in alveolar and interstitial lung macrophages (Fig. 2; Fig. S2, S5A and S6A), extending previous findings (20). IBA1 expression at the leading edge of these cells (Fig. 2F through H) suggests migratory activity. Macrophage overactivation, in particular by syncytial macrophages expressing NRP1 and NRP2 (Fig. 2A and D), may contribute to cytokine storm (57, 58), and syncytia formation is associated with severe disease (59).

Pro-inflammatory pathways transition to fibrosis-related pathways in prolonged COVID-19 (54), with TGFβ playing a key role (60). We detected significant upregulation of the neuropilin ligand *TGFB1* and chemokine *CCL2* in monocytes from fatal COVID-19 cases (Fig. S8B). The significant upregulation of *TGFBR2* in vascular ECs of COVID-19 patients (Fig. S8A) suggests that TGFβ signaling may play a role in the endothelial response to viral infection. This is consistent with previous findings of active TGFβ signaling through TGFBR2 in infected endothelium (35). Endothelial TGFβ signaling increases vascular permeability (61), contributing to vascular leakage and locally compromising the blood-brain barrier in PASC patients, who exhibit elevated serum TGFβ levels (62). Mast cells, also activated by TGFβ (63), express NRP2 (Fig. 4), contain SARS-CoV-2 RNA (Fig. 2N), and are devoid of *ACE2* (Fig. 2K), extending previous findings (20, 30). Mast cell activation has been implicated in PASC pathophysiology (64). Additionally, we observed CD16 (IgG Fc receptor III, FcγIIIb) expression on NRP2-positive mast cells (Fig. 4H), which has been linked to systemic mastocytosis (65). As the mechanism of NRP2 action in chronic mast cell activation is still unclear, further investigation into NRP2 as a potential therapeutic target in PASC is warranted.

### Limitations

The cohorts in this study, comprising COVID-19 patients (*n* = 20) and influenza/noninfectious controls (*n* = 13), were relatively small. NRP2 expression detected by IHC appeared low across samples, with variable intensity likely due to differences in post-mortem intervals. Such variability may introduce noise into the data analysis and obscure biological differences. Further preclinical studies are necessary to evaluate NRP2 modulation as a potential therapeutic approach.

### Conclusion

Combining mRNA and protein expression data *in situ,* we identified NRP1 and NRP2 on cell types exhibiting minimal *ACE2* and *TMPRSS2* expression in the lung, heart, and hematolymphoid organs. In summary, NRP1 was detected in myeloid cells, B lymphocytes, and vascular ECs, while NRP2 expression was largely restricted to macrophages and mast cells. Our study suggests that primary infection with SARS-CoV-2 in the upper respiratory tract depends on ACE2, while neuropilins are important for systemic viral dissemination, monocyte/macrophage-induced vascular damage, microangiopathy, and thrombosis. In this context, neuropilins might play a multifaceted role in SARS-CoV-2 pathogenesis, contributing to both acute and chronic complications. Neuropilins, particularly NRP2, represent promising druggable targets in the treatment of pulmonary fibrosis. As macrophages and mast cells are critically involved in viral spreading, excessive cytokine storm, and fibrotic remodeling of infected tissue, modulation of these cells by targeting NRP2 might offer a new therapeutic strategy in acute COVID-19 as well as in PASC. Further research is necessary to fully understand the functional roles of NRP1 and NRP2 in disease progression.

## MATERIALS AND METHODS

### Patient samples

Autopsy tissues were analyzed using tissue microarrays (TMA) from three different institutions in Germany: University Hospital Bonn (UKB), University Hospital Aachen, and Hannover Medical School (MHH). A total of 20 patients who died from COVID-19 in 2020 were compared with seven patients who succumbed to influenza type H1N1, seasonal A or B. Additionally, six cases of noninfectious deaths were used as controls. The COVID-19 cohort included 11 females and 9 males (mean age: 70.25 ± 11.22 years). The influenza cohort comprised five females and two males (mean age: 50.86 ± 15.24 years), while the uninfected cohort included two females and four males (mean age: 71.25 ± 13.91 years). Infection with SARS-CoV-2 or influenza was confirmed by nasopharyngeal swabs (RT-PCR for SARS-CoV-2 or influenza RNA, respectively). Patient characteristics are provided in Table S1.

### Immunohistochemistry

TMAs of formalin-fixed paraffin-embedded organ samples were assembled using tissue cores 3–4 mm in diameter. Standard paraffin sections (3–4 µm) following standard embedding (fixation: 4% buffered formalin) were used. Morphology was evaluated by hematoxylin-eosin staining. For IHC staining of CD34 and CD68, slides were processed as previously described (66). Serial section staining for CD34 (QBEnd10, Agilent, Santa Clara, USA; 1:200) and CD68 (PG-M1, Agilent; 1:100) was performed using the semiautomatic Autostainer 480S (Medac, Wedel, Germany; for staining conditions see Table S2). Serial section staining for NRP1 (EPR3113, Abcam, Cambridge, UK; 1:200) was performed after deparaffinization with EZ prep (Roche, Basel, Switzerland) and treatment with CC1 buffer (pH 8, Roche) using the automatic BenchMark Ultra staining platform with the OptiView detection kit (Roche; Table S2). TMAs from COVID-19 patients 1–3 (Table S1A) were stained manually in the case of NRP2 with 15 µg/mL (aNRP2-36v2, aTyr Pharma; for protocol see reference 67). Photomicrographs were acquired using either a BX51 microscope (Olympus, Hamburg, Germany) with Zeiss AxioCam MRc5 and Axiovision software (Carl Zeiss, Oberkochen, Germany) or digitally scanned on a Leica scanner (Aperio GT 450 DX, Leica Biosystems, Wetzlar, Germany) and analyzed with QuPath v0.4.3 (68).

For quantification of NRP1-positive myocardial capillaries (Fig. S7), three representative squares in each tissue core were selected in QuPath (each 0.0625 mm^2^) and positive capillaries were counted manually. A mean of three squares was calculated per patient. If multiple cores per patient were available, means were averaged across cores. A total of 35 myocardium samples were analyzed (COVID-19 *n* = 13 patients, influenza *n* = 7 patients, noninfectious controls *n* = 6 patients), and an analysis of variance (ANOVA) was conducted. Insufficient tissue samples were excluded from analysis and staining results confirmed by two independent observers (A.D., I.G.). Variable interpatient NRP1 intensity of staining was observed, possibly reflecting differences in autolytic degradation of autopsy tissue.

Semiquantitative scoring of NRP1 intensity in alveolar macrophages in lungs was conducted in a blinded, independent, and randomized manner. Representative peripheral lung areas per core were photographed in QuPath and evaluated blindly. A total of 56 lung samples were analyzed (COVID-19 *n* = 16 patients, influenza *n* = 7 patients, noninfectious controls *n* = 5 patients). One noninfectious control case (no. 3) was excluded due to lymphangiosis carcinomatosa in the lung. Staining intensities were scored twice independently as follows: 0, negative; 1, low; 2, moderate; and 3, strong (Fig. S2). Discrepant core results were reviewed for a final consensus score.

### CODEX multiplexed tissue imaging

1–3 µm sectioned FFPE TMAs of COVID-19 patients 2 and 3 (Table S1A) were prepared and stained for CODEX-enabled multiplexed tissue imaging following manufacturer’s instructions. The full protocol can be found in (69). A list of antibodies is provided in Table S3. Nuclei were detected using DAPI. Images were analyzed using the Enable Medicine platform (Menlo Park, CA, USA).

### scRNAseq analysis

We re-analyzed a published and annotated scRNAseq data set (30) using preprocessed Python Scanpy objects downloaded from the Single Cell Portal. Data included 19 tissue samples from 18 heart donors (12 males and 6 females) and 24 tissue samples from 16 lung donors with COVID-19 (11 males and 5 females). Clinical metadata and a detailed protocol of tissue collection, tissue procession, RNA extraction, and computational methods for preprocessing, quality control, doublet identification, and dimension reduction are provided in Delorey et al. (2021) (30). Data were normalized using the Scanpy sc.pp.normalize_total function. Annotated doublets and cell clusters containing less than 10 cells were removed. For comparison of COVID-19 lung tissue with healthy tissue samples, Excel files containing log2 fold changes from a single cell-based differential expression model were analyzed (30). All analyzes were performed in Python v3.11.6 (70) with the Scanpy package v1.9.8 (71). The python scripts provided by the original authors on GitHub were modified for re-analysis with focus on genes of interest. Codes are available on GitHub (https://github.com/AlinaDette/Differential-expression-of-Neuropilin-1-NRP1-and-Neuropilin-2-NRP2-in-fatal-COVID-19).

### SARS-CoV-2 RNA detection with fluorescence *in situ* hybridization (FISH)

We performed FISH on 1-µm TMA sections of spleen and lymph node from two COVID-19 cases (patients 2 and 3, Table S1A) with the RNAscope Multiplex Fluorescent Reagent Kit v2 assay (Advanced Cell Diagnostics, Inc., Hayward, CA, USA), following the protocol previously described by reference 31.

### *In vitro* binding experiment

#### Cloning of soluble spike fragments

Cloning of soluble SARS-CoV-2 spike protein fragments was performed by PCR-based site-directed mutagenesis with Phusion Green Hot Start II High-Fidelity (ThermoFisher, Waltham, USA) using pEXPR TO FRT SARS-CoV-2 S d18-SH as template. Mutagenesis primers (Eurofins, Ebersberg, Germany) used in this approach are listed in Table S4. In brief, coding sequences for spike protein fragments S1 and S1′ were generated by introducing a STOP codon at amino acid positions 686 (S1; Ser686Ter) and 816 (S1′; Ser816Ter) and concordantly deleting 3′ sequences of the original spike protein cDNA (NCBI accession number YP_009724390.1). PCR conditions were as follows: 98°C/30 s, 25× (98°C/10 s, 58°C/10 s, 72°C/90 s), 72°C/10 min. PCR fragments were purified using QIAprep Spin Miniprep Kit (Qiagen, Hilden, Germany), A-tailed by incubation with Kapa2G fast HS genotyping mix (Merck, Darmstadt, Germany) in the absence of primers for 20 min at 68°C and cloned into pCR4 using the pCR4 TOPO sequencing kit (Life Technologies, Bleiswijk, Netherlands). Re-cloning into the pCMV6-Entry mammalian expression vector (Origene Technologies, Rockville, USA) used flanking SfaAI and NotI (ThermoFisher) restriction sites. Sequences were verified by Sanger sequencing (Eurofins). To increase secretion, cDNAs for S1 and S1′ fragments were modified by replacing the endogenous by the NRP2 signal peptide in pCMV6-S1 and pCMV6-S1′ (data not shown), followed by a N-terminal hemagglutinin (HA) sequence, using pCMV6-HA-NRP2 as template (72). Primer sequences are listed in Table S4.

#### Production of soluble spike fragments

HEK293 and CaCo-2 cells were obtained from the German Collection of Microorganisms and Cell Cultures (DSMZ), Braunschweig, Germany. HEK293 cells were cultivated in DMEM/F12 media supplemented with 10% fetal bovine serum (FBS) and penicillin/streptomycin (Life Technologies). CaCo-2 cells were grown in MEM/20% FBS and non-essential amino acid supplement. The cells were routinely checked for mycoplasma infection and were free of any contamination. HEK293 and CaCo-2 cDNA were used for real-time quantitative PCR (qRT-PCR) as described in Fig. S3. Expression vectors containing S1 and S1′ fragments were transfected in HEK293 cells using the Effectene transfection method (Qiagen). Stable HEK293 clones were obtained by selection with 400 µg/mL G418 sulfate (Life Technologies). Expression of spike protein fragments in HEK293 clones was determined by Western blotting from cell lysates. Secretion of spike protein fragments was determined from serum-free cell culture supernatant in the presence of 1 mM ACE2 inhibitor (MLN-4760, MedChemExpress, Monmouth Junction, USA) and 10 mM NRP1 inhibitor (EG01377, MedChemExpress) using SARS-CoV-2 spike antibody (ab277628, Abcam, Amsterdam, NL). Secreted spike protein fragments were purified from cell culture supernatants by exploiting their binding to heparin using HiTrap heparin HP affinity columns (Cytiva, Freiburg, Germany) with batch elution with increasing sodium chloride concentration in phosphate buffered saline (pH 7.4, PBS, ThermoFisher). HEK293 cells stably expressing NRP2v2 (RC220706, Origene Technologies) were generated accordingly and checked for expression by Western blot of cell lysates using anti-NRP2 antibody (AF2215, R&D Systems, Abingdon, UK).

### Immunofluorescence

A total of 50,000 cells per 12-mm cover slips were seeded in complete media. After at least 6 h, cells were treated according to the experimental condition and incubated overnight. Immunofluorescence was performed as previously described (73) with the following modifications: cells were fixed on ice for 10 min with 4% formaldehyde in PBS and treated with 1% BSA and 0.5% Triton X-100 in PBS for 20 min prior to incubation with primary and secondary antibodies (Table S5). NRP1 and NRP2 on HEK293 cells were detected using specific antibodies (EPR3113 and EPR23808-72, respectively; Abcam Ltd., Cambridge, UK). HEK293 cells not expressing the NRP2 construct were used as internal negative controls for binding specificity. C29F4 antibody (Cell Signaling Technology, Leiden, Netherlands) was used for HA-tagged spike protein fragments. Rabbit isotype controls were purchased from Cell Signaling Technology. Controls are depicted in Fig. S4. Cell membrane/F-actin staining was performed using phalloidin (A30104, Thermo Fisher Scientific, Langerwehe, Germany) and cells were embedded using Fluoromount-G mounting medium with DAPI (Thermo Fisher Scientific).

## Data Availability

All data in this study and the analysis code are available from the corresponding author (I. Gütgemann) upon request. scRNAseq data were downloaded from the Single Cell Portal, as follows: heart, https://singlecell.broadinstitute.org/single_cell/study/SCP1216/, and lung, https://singlecell.broadinstitute.org/single_cell/study/SCP1052/.

## SUPPLEMENTAL MATERIAL

Supplemental material jvi.01384-25-s0001.pdf Tables S1 to S5; Fig. S1 to S8.

ASM does not own the copyrights to Supplemental Material that may be linked to, or accessed through, an article. The authors have granted ASM a non-exclusive, world-wide license to publish the Supplemental Material files. Please contact the corresponding author directly for reuse.

## References

[1] Jing H, Wu X, Xiang M, Liu L, Novakovic VA, Shi J. 2022. Pathophysiological mechanisms of thrombosis in acute and long COVID-19. Front Immunol 13:992384. doi:10.3389/fimmu.2022.992384 36466841 PMC9709252

[2] Wrapp D, Wang N, Corbett KS, Goldsmith JA, Hsieh C-L, Abiona O, Graham BS, McLellan JS. 2020. Cryo-EM structure of the 2019-nCoV spike in the prefusion conformation. Science 367:1260–1263. doi:10.1126/science.abb2507 32075877 PMC7164637

[3] Jafarzadeh A, Chauhan P, Saha B, Jafarzadeh S, Nemati M. 2020. Contribution of monocytes and macrophages to the local tissue inflammation and cytokine storm in COVID-19: Lessons from SARS and MERS, and potential therapeutic interventions. Life Sci 257:118102. doi:10.1016/j.lfs.2020.118102 32687918 PMC7367812

[4] Schulte-Schrepping J, Reusch N, Paclik D, Baßler K, Schlickeiser S, Zhang B, Krämer B, Krammer T, Brumhard S, Bonaguro L, et al.. 2020. Severe COVID-19 is marked by a dysregulated myeloid cell compartment. Cell 182:1419–1440. doi:10.1016/j.cell.2020.08.001 32810438 PMC7405822

[5] Iba T, Levy JH, Maier CL, Connors JM, Levi M. 2024. Four years into the pandemic, managing COVID-19 patients with acute coagulopathy: what have we learned? J Thromb Haemost 22:1541–1549. doi:10.1016/j.jtha.2024.02.013 38428590

[6] Proal AD, VanElzakker MB, Aleman S, Bach K, Boribong BP, Buggert M, Cherry S, Chertow DS, Davies HE, Dupont CL, et al.. 2023. SARS-CoV-2 reservoir in post-acute sequelae of COVID-19 (PASC). Nat Immunol 24:1778–1778. doi:10.1038/s41590-023-01601-2 37667052

[7] Davis HE, McCorkell L, Vogel JM, Topol EJ. 2023. Long COVID: major findings, mechanisms and recommendations. Nat Rev Microbiol 21:133–146. doi:10.1038/s41579-022-00846-2 36639608 PMC9839201

[8] Alrajhi NN. 2023. Post-COVID-19 pulmonary fibrosis: an ongoing concern. Ann Thorac Med 18:173–181. doi:10.4103/atm.atm_7_23 38058790 PMC10697304

[9] Jackson CB, Farzan M, Chen B, Choe H. 2022. Mechanisms of SARS-CoV-2 entry into cells. Nat Rev Mol Cell Biol 23:3–20. doi:10.1038/s41580-021-00418-x 34611326 PMC8491763

[10] Li M-Y, Li L, Zhang Y, Wang X-S. 2020. Expression of the SARS-CoV-2 cell receptor gene ACE2 in a wide variety of human tissues. Infect Dis Poverty 9:45. doi:10.1186/s40249-020-00662-x 32345362 PMC7186534

[11] Zhou L, Niu Z, Jiang X, Zhang Z, Zheng Y, Wang Z, Zhu Y, Gao L, Huang H, Wang X, Sun Q. 2020. SARS-CoV-2 targets by the pscRNA profiling of ACE2, TMPRSS2 and furin proteases. iScience 23:101744. doi:10.1016/j.isci.2020.101744 33134888 PMC7591870

[12] Lindskog C, Méar L, Virhammar J, Fällmar D, Kumlien E, Hesselager G, Casar-Borota O, Rostami E. 2022. Protein expression profile of ACE2 in the normal and COVID-19-affected human brain. J Proteome Res 21:2137–2145. doi:10.1021/acs.jproteome.2c00184 35901083 PMC9364976

[13] Cantuti-Castelvetri L, Ojha R, Pedro LD, Djannatian M, Franz J, Kuivanen S, van der Meer F, Kallio K, Kaya T, Anastasina M, et al.. 2020. Neuropilin-1 facilitates SARS-CoV-2 cell entry and infectivity. Science 370:856–860. doi:10.1126/science.abd2985 33082293 PMC7857391

[14] McCracken IR, Saginc G, He L, Huseynov A, Daniels A, Fletcher S, Peghaire C, Kalna V, Andaloussi-Mäe M, Muhl L, Craig NM, Griffiths SJ, Haas JG, Tait-Burkard C, Lendahl U, Birdsey GM, Betsholtz C, Noseda M, Baker AH, Randi AM. 2021. Lack of evidence of angiotensin-converting enzyme 2 expression and replicative infection by SARS-CoV-2 in human endothelial cells. Circulation 143:865–868. doi:10.1161/CIRCULATIONAHA.120.052824 33405941 PMC7899720

[15] Daly JL, Simonetti B, Klein K, Chen K-E, Williamson MK, Antón-Plágaro C, Shoemark DK, Simón-Gracia L, Bauer M, Hollandi R, Greber UF, Horvath P, Sessions RB, Helenius A, Hiscox JA, Teesalu T, Matthews DA, Davidson AD, Collins BM, Cullen PJ, Yamauchi Y. 2020. Neuropilin-1 is a host factor for SARS-CoV-2 infection. Science 370:861–865. doi:10.1126/science.abd3072 33082294 PMC7612957

[16] Hoffmann M, Kleine-Weber H, Schroeder S, Krüger N, Herrler T, Erichsen S, Schiergens TS, Herrler G, Wu N-H, Nitsche A, Müller MA, Drosten C, Pöhlmann S. 2020. SARS-CoV-2 cell entry depends on ACE2 and TMPRSS2 and is blocked by a clinically proven protease inhibitor. Cell 181:271–280. doi:10.1016/j.cell.2020.02.052 32142651 PMC7102627

[17] Teesalu T, Sugahara KN, Kotamraju VR, Ruoslahti E. 2009. C-end rule peptides mediate neuropilin-1-dependent cell, vascular, and tissue penetration. Proc Natl Acad Sci USA 106:16157–16162. doi:10.1073/pnas.0908201106 19805273 PMC2752543

[18] Ishitoku M, Mokuda S, Araki K, Watanabe H, Kohno H, Sugimoto T, Yoshida Y, Sakaguchi T, Masumoto J, Hirata S, Sugiyama E. 2023. Tumor necrosis factor and interleukin-1β upregulate NRP2 expression and promote SARS-CoV-2 proliferation. Viruses 15:1498. doi:10.3390/v15071498 37515185 PMC10383177

[19] Balistreri G, Yamauchi Y, Teesalu T. 2021. A widespread viral entry mechanism: the C-end Rule motif-neuropilin receptor interaction. Proc Natl Acad Sci USA 118:e2112457118. doi:10.1073/pnas.2112457118 34772761 PMC8670474

[20] Roy S, Bag AK, Singh RK, Talmadge JE, Batra SK, Datta K. 2017. Multifaceted role of neuropilins in the immune system: potential targets for immunotherapy. Front Immunol 8:1228. doi:10.3389/fimmu.2017.01228 29067024 PMC5641316

[21] Schellenburg S, Schulz A, Poitz DM, Muders MH. 2017. Role of neuropilin-2 in the immune system. Mol Immunol 90:239–244. doi:10.1016/j.molimm.2017.08.010 28843905

[22] Islam R, Mishra J, Bodas S, Bhattacharya S, Batra SK, Dutta S, Datta K. 2022. Role of neuropilin-2-mediated signaling axis in cancer progression and therapy resistance. Cancer Metastasis Rev 41:771–787. doi:10.1007/s10555-022-10048-0 35776228 PMC9247951

[23] Chekol Abebe E, Mengie Ayele T, Tilahun Muche Z, Asmamaw Dejenie T. 2021. Neuropilin 1: a novel entry factor for SARS-CoV-2 infection and a potential therapeutic target. Biol Targets Ther 15:143–152. doi:10.2147/BTT.S307352 PMC8110213 33986591

[24] Wu C-T, Lidsky PV, Xiao Y, Lee IT, Cheng R, Nakayama T, Jiang S, Demeter J, Bevacqua RJ, Chang CA, Whitener RL, Stalder AK, Zhu B, Chen H, Goltsev Y, Tzankov A, Nayak JV, Nolan GP, Matter MS, Andino R, Jackson PK. 2021. SARS-CoV-2 infects human pancreatic β cells and elicits β cell impairment. Cell Metab 33:1565–1576. doi:10.1016/j.cmet.2021.05.013 34081912 PMC8130512

[25] Assous M, Martinez E, Eisenberg C, Shah F, Kosc A, Varghese K, Espinoza D, Bhimani S, Tepper JM, Shiflett MW, Tran TS. 2019. Neuropilin 2 signaling mediates corticostriatal transmission, spine maintenance, and goal-directed learning in mice. J Neurosci 39:8845–8859. doi:10.1523/JNEUROSCI.1006-19.2019 31541021 PMC6832683

[26] Lambrinos G, Cristofaro V, Pelton K, Bigger-Allen A, Doyle C, Vasquez E, Bielenberg DR, Sullivan MP, Adam RM. 2022. Neuropilin 2 is a novel regulator of distal colon contractility. Am J Pathol 192:1592–1603. doi:10.1016/j.ajpath.2022.07.013 35985479 PMC9667714

[27] Ackermann M, Verleden SE, Kuehnel M, Haverich A, Welte T, Laenger F, Vanstapel A, Werlein C, Stark H, Tzankov A, Li WW, Li VW, Mentzer SJ, Jonigk D. 2020. Pulmonary vascular endothelialitis, thrombosis, and angiogenesis in Covid-19. N Engl J Med 383:120–128. doi:10.1056/NEJMoa2015432 32437596 PMC7412750

[28] Sarabipour S, Mac Gabhann F. 2021. Targeting neuropilins as a viable SARS-CoV-2 treatment. FEBS J 288:5122–5129. doi:10.1111/febs.16096 34185437 PMC8420456

[29] Goltsev Y, Samusik N, Kennedy-Darling J, Bhate S, Hale M, Vazquez G, Black S, Nolan GP. 2018. Deep profiling of mouse splenic architecture with CODEX multiplexed imaging. Cell 174:968–981. doi:10.1016/j.cell.2018.07.010 30078711 PMC6086938

[30] Delorey TM, Ziegler CGK, Heimberg G, Normand R, Yang Y, Segerstolpe Å, Abbondanza D, Fleming SJ, Subramanian A, Montoro DT, et al.. 2021. COVID-19 tissue atlases reveal SARS-CoV-2 pathology and cellular targets. Nature 595:107–113. doi:10.1038/s41586-021-03570-8 33915569 PMC8919505

[31] Wong DWL, Klinkhammer BM, Djudjaj S, Villwock S, Timm MC, Buhl EM, Wucherpfennig S, Cacchi C, Braunschweig T, Knüchel-Clarke R, Jonigk D, Werlein C, Bülow RD, Dahl E, von Stillfried S, Boor P. 2021. Multisystemic cellular tropism of SARS-CoV-2 in autopsies of COVID-19 patients. Cells 10:1900. doi:10.3390/cells10081900 34440669 PMC8394956

[32] Werlein C, Ackermann M, Stark H, Shah HR, Tzankov A, Haslbauer JD, von Stillfried S, Bülow RD, El-Armouche A, Kuenzel S, et al.. 2023. Inflammation and vascular remodeling in COVID-19 hearts. Angiogenesis 26:233–248. doi:10.1007/s10456-022-09860-7 36371548 PMC9660162

[33] Chen L, Li X, Chen M, Feng Y, Xiong C. 2020. The ACE2 expression in human heart indicates new potential mechanism of heart injury among patients infected with SARS-CoV-2. Cardiovasc Res 116:1097–1100. doi:10.1093/cvr/cvaa078 32227090 PMC7184507

[34] Ohsawa K, Imai Y, Kanazawa H, Sasaki Y, Kohsaka S. 2000. Involvement of Iba1 in membrane ruffling and phagocytosis of macrophages/microglia. J Cell Sci 113:3073–3084. doi:10.1242/jcs.113.17.3073 10934045

[35] Zhao G, Xue L, Weiner AI, Gong N, Adams-Tzivelekidis S, Wong J, Gentile ME, Nottingham AN, Basil MC, Lin SM, Niethamer TK, Diamond JM, Bermudez CA, Cantu E, Han X, Cao Y, Alameh M-G, Weissman D, Morrisey EE, Mitchell MJ, Vaughan AE. 2024. TGF-βR2 signaling coordinates pulmonary vascular repair after viral injury in mice and human tissue. Sci Transl Med 16:eadg6229. doi:10.1126/scitranslmed.adg6229 38295183 PMC12067352

[36] Gharaee-Kermani M, Denholm EM, Phan SH. 1996. Costimulation of fibroblast collagen and transforming growth factor β1 gene expression by monocyte chemoattractant protein-1 via specific receptors. J Biol Chem 271:17779–17784. doi:10.1074/jbc.271.30.17779 8663511

[37] Venet M, Ribeiro MS, Décembre E, Bellomo A, Joshi G, Nuovo C, Villard M, Cluet D, Perret M, Pescamona R, Paidassi H, Walzer T, Allatif O, Belot A, Trouillet-Assant S, Ricci EP, Dreux M. 2023. Severe COVID-19 patients have impaired plasmacytoid dendritic cell-mediated control of SARS-CoV-2. Nat Commun 14:694. doi:10.1038/s41467-023-36140-9 36755036 PMC9907212

[38] Radzikowska U, Ding M, Tan G, Zhakparov D, Peng Y, Wawrzyniak P, Wang M, Li S, Morita H, Altunbulakli C, Reiger M, Neumann AU, Lunjani N, Traidl-Hoffmann C, Nadeau KC, O’Mahony L, Akdis C, Sokolowska M. 2020. Distribution of ACE2, CD147, CD26, and other SARS-CoV-2 associated molecules in tissues and immune cells in health and in asthma, COPD, obesity, hypertension, and COVID-19 risk factors. Allergy 75:2829–2845. doi:10.1111/all.14429 32496587 PMC7300910

[39] Pontelli MC, Castro ÍA, Martins RB, La Serra L, Veras FP, Nascimento DC, Silva CM, Cardoso RS, Rosales R, Gomes R, et al.. 2022. SARS-CoV-2 productively infects primary human immune system cells in vitro and in COVID-19 patients. J Mol Cell Biol 14:mjac021. doi:10.1093/jmcb/mjac021 35451490 PMC9384834

[40] Huang I, Pranata R. 2020. Lymphopenia in severe coronavirus disease-2019 (COVID-19): systematic review and meta-analysis. J Intensive Care 8:36. doi:10.1186/s40560-020-00453-4 32483488 PMC7245646

[41] Bruder D, Probst-Kepper M, Westendorf AM, Geffers R, Beissert S, Loser K, von Boehmer H, Buer J, Hansen W. 2004. Neuropilin-1: a surface marker of regulatory T cells. Eur J Immunol 34:623–630. doi:10.1002/eji.200324799 14991591

[42] Haunhorst S, Bloch W, Javelle F, Krüger K, Baumgart S, Drube S, Lemhöfer C, Reuken P, Stallmach A, Müller M, Zielinski CE, Pletz MW, Gabriel HHW, Puta C. 2022. A scoping review of regulatory T cell dynamics in convalescent COVID-19 patients - indications for their potential involvement in the development of Long COVID? Front Immunol 13:1070994. doi:10.3389/fimmu.2022.1070994 36582234 PMC9792979

[43] Yin K, Peluso MJ, Luo X, Thomas R, Shin M-G, Neidleman J, Andrew A, Young KC, Ma T, Hoh R, et al.. 2024. Long COVID manifests with T cell dysregulation, inflammation and an uncoordinated adaptive immune response to SARS-CoV-2. Nat Immunol 25:218–225. doi:10.1038/s41590-023-01724-6 38212464 PMC10834368

[44] Hou D, Cao W, Kim S, Cui X, Ziarnik M, Im W, Zhang XF. 2023. Biophysical investigation of interactions between SARS-CoV-2 spike protein and neuropilin-1. Protein Sci 32:e4773. doi:10.1002/pro.4773 37656811 PMC10510470

[45] Husain B, Yuen K, Sun D, Cao S, Payandeh J, Martinez-Martin N. 2022. Cell-based receptor discovery identifies host factors specifically targeted by the SARS CoV-2 spike. Commun Biol 5:788. doi:10.1038/s42003-022-03695-0 35931765 PMC9355963

[46] Parker MW, Linkugel AD, Goel HL, Wu T, Mercurio AM, Vander Kooi CW. 2015. Structural basis for VEGF-C binding to neuropilin-2 and sequestration by a soluble splice form. Structure 23:677–687. doi:10.1016/j.str.2015.01.018 25752543 PMC4394031

[47] Johnson BA, Xie X, Bailey AL, Kalveram B, Lokugamage KG, Muruato A, Zou J, Zhang X, Juelich T, Smith JK, et al.. 2021. Loss of furin cleavage site attenuates SARS-CoV-2 pathogenesis. Nature 591:293–299. doi:10.1038/s41586-021-03237-4 33494095 PMC8175039

[48] Frolova EI, Palchevska O, Lukash T, Dominguez F, Britt W, Frolov I. 2022. Acquisition of furin cleavage site and further SARS-CoV-2 evolution change the mechanisms of viral entry, infection spread, and cell signaling. J Virol 96:e0075322. doi:10.1128/jvi.00753-22 35876526 PMC9364789

[49] Xu S-W, Ilyas I, Weng J-P. 2023. Endothelial dysfunction in COVID-19: an overview of evidence, biomarkers, mechanisms and potential therapies. Acta Pharmacol Sin 44:695–709. doi:10.1038/s41401-022-00998-0 36253560 PMC9574180

[50] Hikmet F, Méar L, Edvinsson Å, Micke P, Uhlén M, Lindskog C. 2020. The protein expression profile of ACE2 in human tissues. Mol Syst Biol 16:e9610. doi:10.15252/msb.20209610 32715618 PMC7383091

[51] Shweiki D, Itin A, Soffer D, Keshet E. 1992. Vascular endothelial growth factor induced by hypoxia may mediate hypoxia-initiated angiogenesis. Nature 359:843–845. doi:10.1038/359843a0 1279431

[52] Barbosa LC, Gonçalves TL, de Araujo LP, Rosario LV de O, Ferrer VP. 2021. Endothelial cells and SARS-CoV-2: an intimate relationship. Vascul Pharmacol 137:106829. doi:10.1016/j.vph.2021.106829 33422689 PMC7834309

[53] Fantin A, Vieira JM, Plein A, Denti L, Fruttiger M, Pollard JW, Ruhrberg C. 2013. NRP1 acts cell autonomously in endothelium to promote tip cell function during sprouting angiogenesis. Blood 121:2352–2362. doi:10.1182/blood-2012-05-424713 23315162 PMC3606070

[54] Ackermann M, Kamp JC, Werlein C, Walsh CL, Stark H, Prade V, Surabattula R, Wagner WL, Disney C, Bodey AJ, et al.. 2022. The fatal trajectory of pulmonary COVID-19 is driven by lobular ischemia and fibrotic remodelling. EBioMedicine 85:104296. doi:10.1016/j.ebiom.2022.104296 36206625 PMC9535314

[55] Dhupar R, Powers AA, Eisenberg SH, Gemmill RM, Bardawil CE, Udoh HM, Cubitt A, Nangle LA, Soloff AC. 2024. Orchestrating resilience: how neuropilin-2 and macrophages contribute to cardiothoracic disease. J Clin Med 13:1446. doi:10.3390/jcm13051446 38592275 PMC10934188

[56] Vosbeck K, Förster S, Mayr T, Sahu A, Haddouti E-M, Al-Adilee O, Körber R-M, Bisht S, Muders MH, Nesic S, Buness A, Kristiansen G, Schildberg FA, Gütgemann I. 2024. Neuropilin2 in mesenchymal stromal cells as a potential novel therapeutic target in myelofibrosis. Cancers (Basel) 16:1924. doi:10.3390/cancers16101924 38792002 PMC11119673

[57] Felkle D, Zięba K, Kaleta K, Czaja J, Zyzdorf A, Sobocińska W, Jarczyński M, Bryniarski K, Nazimek K. 2023. Overreactive macrophages in SARS-CoV-2 infection: the effects of ACEI. Int Immunopharmacol 124:110858. doi:10.1016/j.intimp.2023.110858 37708705

[58] Milde R, Ritter J, Tennent GA, Loesch A, Martinez FO, Gordon S, Pepys MB, Verschoor A, Helming L. 2015. Multinucleated giant cells are specialized for complement-mediated phagocytosis and large target destruction. Cell Rep 13:1937–1948. doi:10.1016/j.celrep.2015.10.065 26628365 PMC4675895

[59] Rajah MM, Bernier A, Buchrieser J, Schwartz O. 2022. The mechanism and consequences of SARS-CoV-2 spike-mediated fusion and syncytia formation. J Mol Biol 434:167280. doi:10.1016/j.jmb.2021.167280 34606831 PMC8485708

[60] Zhang K, Phan SH. 1996. Cytokines and pulmonary fibrosis. Biol Signals 5:232–239. doi:10.1159/000109195 8891199

[61] Lee YH, Kayyali US, Sousa AM, Rajan T, Lechleider RJ, Day RM. 2007. Transforming growth factor-β1 effects on endothelial monolayer permeability involve focal adhesion kinase/Src. Am J Respir Cell Mol Biol 37:485–493. doi:10.1165/rcmb.2006-0439OC 17585111 PMC2176121

[62] Greene C, Connolly R, Brennan D, Laffan A, O’Keeffe E, Zaporojan L, O’Callaghan J, Thomson B, Connolly E, Argue R, Meaney JFM, Martin-Loeches I, Long A, Cheallaigh CN, Conlon N, Doherty CP, Campbell M. 2024. Blood-brain barrier disruption and sustained systemic inflammation in individuals with long COVID-associated cognitive impairment. Nat Neurosci 27:421–432. doi:10.1038/s41593-024-01576-9 38388736 PMC10917679

[63] Haque TT, Frischmeyer-Guerrerio PA. 2022. The role of TGFβ and other cytokines in regulating mast cell functions in allergic inflammation. Int J Mol Sci 23:10864. doi:10.3390/ijms231810864 36142776 PMC9503477

[64] Sumantri S, Rengganis I. 2023. Immunological dysfunction and mast cell activation syndrome in long COVID. Asia Pac Allergy 13:50–53. doi:10.5415/apallergy.0000000000000022 37389095 PMC10166245

[65] Teodosio C, Mayado A, Sánchez-Muñoz L, Morgado JM, Jara-Acevedo M, Álvarez-Twose I, García-Montero AC, Matito A, Caldas C, Escribano L, Orfao A. 2015. The immunophenotype of mast cells and its utility in the diagnostic work-up of systemic mastocytosis. J Leukoc Biol 97:49–59. doi:10.1189/jlb.5RU0614-296R 25381388

[66] Koerber R-M, Schneider RK, Pritchard JE, Teichmann LL, Schumacher U, Brossart P, Gütgemann I. 2023. Nestin expression in osteocytes following myeloablation and during bone marrow metastasis. Br J Haematol 200:643–651. doi:10.1111/bjh.18563 36382360

[67] Förster S, Chong YE, Siefker D, Becker Y, Bao R, Escobedo E, Qing Y, Rauch K, Burman L, Burkart C, Kainz P, Cubitt A, Muders M, Nangle LA. 2023. Development and characterization of a novel neuropilin-2 antibody for immunohistochemical staining of cancer and sarcoidosis tissue samples. Monoclon Antib Immunodiagn Immunother 42:157–165. doi:10.1089/mab.2023.0007 37902990

[68] Bankhead P, Loughrey MB, Fernández JA, Dombrowski Y, McArt DG, Dunne PD, McQuaid S, Gray RT, Murray LJ, Coleman HG, James JA, Salto-Tellez M, Hamilton PW. 2017. QuPath: open source software for digital pathology image analysis. Sci Rep 7:16878. doi:10.1038/s41598-017-17204-5 29203879 PMC5715110

[69] Black S, Phillips D, Hickey JW, Kennedy-Darling J, Venkataraaman VG, Samusik N, Goltsev Y, Schürch CM, Nolan GP. 2021. CODEX multiplexed tissue imaging with DNA-conjugated antibodies. Nat Protoc 16:3802–3835. doi:10.1038/s41596-021-00556-8 34215862 PMC8647621

[70] van RG, Drake FL. 2010. The Python language reference Release 3.0.1 [Repr.]. Python Software Foundation, Hampton, NH.

[71] Wolf FA, Angerer P, Theis FJ. 2018. SCANPY: large-scale single-cell gene expression data analysis. Genome Biol 19:15. doi:10.1186/s13059-017-1382-0 29409532 PMC5802054

[72] Dutta S, Polavaram NS, Islam R, Bhattacharya S, Bodas S, Mayr T, Roy S, Albala SAY, Toma MI, Darehshouri A, Borkowetz A, Conrad S, Fuessel S, Wirth M, Baretton GB, Hofbauer LC, Ghosh P, Pienta KJ, Klinkebiel DL, Batra SK, Muders MH, Datta K. 2022. Neuropilin-2 regulates androgen-receptor transcriptional activity in advanced prostate cancer. Oncogene 41:3747–3760. doi:10.1038/s41388-022-02382-y 35754042 PMC9979947

[73] Becker Y, Förster S, Gielen GH, Loke I, Thaysen-Andersen M, Laurini C, Wehrand K, Pietsch T, Diestel S. 2019. Paucimannosidic glycoepitopes inhibit tumorigenic processes in glioblastoma multiforme. Oncotarget 10:4449–4465. doi:10.18632/oncotarget.27056 31320997 PMC6633888

