# Supplementary material for: Differential expression of viral entry protein neuropilin 1 (NRP1) and neuropilin 2 (NRP2) in fatal COVID-19
Source: J Virol. 2025 Oct 29;99(11):e01384-25. doi: 10.1128/jvi.01384-25 (PMC12645918; doi:10.1128/jvi.01384-25)
Supplement: Supplemental material — Tables S1 to S5; Fig. S1 to S8. [file jvi.01384-25-s0001.pdf]

Supplement to: Dette A., Moers F., Mayr T. et al. Differential expression of viral entry protein Neuropilin 1 (NRP1) and Neuropilin 2 (NRP2) in fatal COVID-19

Table of Contents

Supplementary Tables

Table S1. Overview of clinical cohort 2

Table S2. Antibodies and titration, immunohistochemistry (IHC) 6

Table S3. Staining antibodies for Co-detection by indexing (CODEX) 6

Table S4. Primers 7

Table S5. Antibodies, immunofluorescence 9

Supplementary Figures

Figure S1. Schematic diagram of SARS-CoV-2 entry into host cells 10

Figure S2. Semiquantitative analysis of NRP1 (IHC), lung 11

Figure S3. Relative gene expression analysis ($\Delta\Delta C\tau$) in HEK293 cells and NRP2-transfected HEK293 cells 12

Figure S4. Immunofluorescence (IF) of HEK293 cells 13

Figure S5. NRP1 expression in fatal COVID-19 (IHC), various organs 14

Figure S6. NRP2 expression in fatal COVID-19 (IHC), various organs 15

Figure S7. Quantitative analysis of NRP1 (IHC), heart 16

Figure S8. Differential gene expression in fatal COVID-19 versus non-infectious controls, lung 17

References 18

Pat ID	Age (years)	Gender	BMI	Smoking	Diabetes Type 2	Hypertension	Immuno-suppression	COPD or asthma	Hospital Admission to Death (days)	Death to autopsy (hours)	Ventilation	Duration of Ventilation Therapy (days)	ECMO	virus detection (PCR)	Weight of heart (gram)	weight of lung (gram)	weight of spleen (gram)	Cause of death
1	71-75	f	27	n.a.	yes	yes	no	no	7	45	NIV, invasive	7	no	yes (lung, heart)	459	1581	261	DAD/ARDS
2	71-75	f	27	n.a.	no	no	no	no	33	42	vv-ECMO	32	yes	yes (lung, trachea)	392	1472	208	DAD/ARDS
3	56-60	m	25	n.a.	yes	yes	no	no	24	24	BIPAP	24	yes	yes (lung, trachea, heart)	639	2121	289	DAD/ARDS
4	76-80	m	32	n.a.	no	no	no	no	2	29	-	0	no	yes (lung, heart)	502	2350	206	DAD/ARDS
5	66-70	m	29	n.a.	no	no	no	no	29	30	NO inhalation	29	no	yes (lung, trachea, heart)	751	3984	624	DAD/ARDS
6	61-65	f	24	n.a.	no	no	yes	no	63	48	invasive	57	no	yes (lung, trachea)	396	1487	230	DAD/ARDS
7	51-55	m	23	n.a.	n.a.	n.a.	n.a.	n.a.	45	41	vv-ECMO	44	yes	yes (lung, heart)	461	1646	240	sepsis, MOF
8	51-55	f	41	n.a.	no	yes	no	asthma	16	28	NIV/CPAP vv-ECMO	n.a.	yes	yes (lung, heart)	385	1480	255	myocardial infarction
9	91-95	m	18	no	no	yes	no	n.a.	14	n.a.	-	0	no	n.a.	-	n.a.	-	septic MOF due to pneumonia
10	81-85	f	n.a.	yes	yes	no	no	n.a.	12	n.a.	invasive	n.a.	no	n.a.	-	n.a.	-	pneumonia with MOF
11	76-80	m	31	n.a.	no	yes	no	n.a.	22	n.a.	PCV	22	no	n.a.	-	n.a.	-	pneumonia with MOF

12	71-75	m	24	n.a.	no	yes	yes	n.a.	6	n.a.	invasive	n.a.	no	n.a.	n.a.	-	-	MOF, rhabdomyo-lysis
13	71-75	m	43	n.a.	yes	yes	no	n.a.	12	n.a.	PCV	12	no	n.a.	-	n.a.	-	RF, septic MOF
14	66-70	f	n.a.	n.a.	no	yes	no	n.a.	14	n.a.	PCV	13	no	n.a.	-	n.a.	-	n.a.
15	76-80	f	n.a.	n.a.	yes	no	no	n.a.	7	n.a.	PCV	5	no	n.a.	-	n.a.	-	n.a.
16	51-55	m	n.a.	n.a.	n.a.	n.a.	n.a.	n.a.	21	n.a.	n.a.	n.a.	no	n.a.	-	n.a.	-	RF
17	76-80	f	36	no	yes	no	no	n.a.	14	n.a.	invasive	n.a.	no	n.a.	n.a.	-	-	ARDS, septic shock, MOF
18	71-75	f	26	n.a.	yes	no	yes	n.a.	5	n.a.	invasive	n.a.	no	n.a.	n.a.	-	-	RF
19	76-80	f	n.a.	n.a.	n.a.	n.a.	n.a.	n.a.	23	n.a.	n.a.	n.a.	n.a.	n.a.	n.a.	-	-	n.a.
20	56-60	f	34	n.a.	no	yes	no	n.a.	31	48	invasive	24	yes	n.a.	545	2152	465	CRF

Table S1A. Overview of clinical cohort: COVID-19 patients' characteristics. Patients 1–8 came from the University Hospital Aachen, patients 9–19 from Hannover Medical School and patient 20 from University Hospital Bonn. BMI = body-mass-index, COPD = chronic obstructive pulmonary disease, PCV = pressure-controlled ventilation, BIPAP = bilevel positive airway pressure, CPAP = continuous positive airway pressure, DAD = diffuse alveolar damage, ARDS = acute respiratory distress syndrome, CRF = cardio-respiratory failure, vv-ECMO = veno-venous extracorporeal membrane oxygenation, MOF = multi-organ-failure, RF = respiratory failure, n.a. = not available

Pat ID	Age	Gender	BMI	Smoking	Diabetes Type 2	Hypertension	Immuno-suppression	COPD or asthma	Hospital Admission to Death (days)	Death to autopsy (h)	Ventilation	Duration of Ventilation Therapy (days)	ECMO	Influenza subtype	Weight of heart (gram)	Weight of lung (gram)	Weight of spleen (gram)	Cause of death
1	31-35	f	28	n.a.	no	no	no	no	3	31	vv-ECMO	n.a.	yes	H1N1	293	n.a.	n.a.	DAD/ARDS
2	51-55	m	42	n.a.	no	yes	no	no	14	13	vv-ECMO	13	yes	H1N1	568	2534	423	sepsis, MOF
3	31-35	f	32	yes	no	yes	no	no	10	36	vv-ECMO	16	yes	seasonal type B	435	1814	364	sepsis, MOF
4	71-75	f	26	n.a.	no	yes	no	no	16	18	n.a.	3	no	seasonal type B	300	1005	168	sepsis, MOF
5	41-45	f	27	n.a.	no	no	no	no	23	12	n.a.	n.a.	no	H1N1	348	1700	205	sepsis, MOF
6	56-60	m	37	yes	no	yes	no	no	6	12	NIV, invasive	4	no	H1N1	840	2510	222	DAD/ARDS
7	61-65	f	21	n.a.	no	no	yes	no	16	34	no	0	n.a.	seasonal type A	350	1200	300	cardiac arrhythmogenic event due to sepsis

Table S1B. Overview of clinical cohort: Influenza patients' characteristics. All patients came from University Hospital Aachen. BMI = body-mass-index, COPD = chronic obstructive pulmonary disease, NIV = non-invasive ventilation, DAD = diffuse alveolar damage, ARDS = acute respiratory distress syndrome, vv-ECMO = veno-venous extracorporeal membrane oxygenation, MOF = multi-organ-failure, n.a. = not available

Pat ID	Age	Gender	BMI	Smoking	Diabetes Type 2	Hypertension	Immuno-suppression	COPD or asthma	Hospital Admission to Death (days)	Death to autopsy (h)	Ventilation	Duration of Ventilation Therapy (days)	ECMO	Weight of heart (gram)	Weight of lung (gram)	Weight of spleen (gram)	Cause of death
1	56-60	f	21	n.a.	no	no	yes	no	41	24	-	0	no	435	1097	286	NHL with MOF
2	86-90	m	25	n.a.	no	no	no	no	-	30	-	0	no	385	1463	69	cardiac tamponade
3	71-75	f	24	n.a.	no	no	no	no	-	36	-	0	no	257	1053	65	central dysregulation due to brain metastases (NSCLC)
4	85-90	m	21	yes	no	yes	no	no	-	68	-	0	no	548	2160 (after formalin)	270	sepsis due to sacral ulcer
5	61-65	m	30	n.a.	no	yes	no	COPD	-	23	-	0	no	600	1738	360	mesenterial ischemia with MOF
6	56-60	m	27	n.a.	no	no	no	no	-	65	-	0	no	1020	1551	323	dilated cardiomyopathy

Table S1C. Overview of clinical cohort: Noninfectious control patients' characteristics. All patients came from University Hospital Aachen. BMI = body-mass-index, COPD = chronic obstructive pulmonary disease, NIV = non-invasive ventilation, MOF = multi-organ-failure, NHL = non-hodgkin lymphoma, MOF = multi-organ failure, NSCLC = non-small-cell lung cancer, n.a. = not available

Antigen	Clone	Dilution	Retrieval	Retrieval time, temperature	Incubation of antibody	Catalogue no.	Manufacturer	Staining platform
NRP1	EPR3113	1:200	CC1 pH 8	48 min, 95°C	36 min	ab184784	Abcam	BenchMark Ultra
CD34	QBEnd10	1:200	Citrate buffer pH 6	20 min, 99°C	30 min	M7165	Agilent	Autostainer 480-S
CD68	PG-M1	1:100	Citrate buffer pH 6	20 min, 99°C	30 min	M0876	Agilent	Autostainer 480-S

Table S2. Antibodies and titration, immunohistochemistry (IHC).

Marker	Clone	Source	Barcode	Dilution
CD31	EP3095	Akoya	BX001	1:100
CD4	EPR6855	Akoya	BX003	1:200
CD20	L26	Akoya	BX007	1:200
CD68	KP1	Akoya	BX015	1:200
PanCK	AE-1	Akoya	BX019	1:200
CD8	C8/144B	Akoya	BX026	1:200
HLA-DR	EPR3692	Akoya	BX033	1:200

NRP2	aNRP2-36v2	aTyr Pharma	BX017	1:100
Mast Cell	AA1	Abcam	BX028	1:1500
NRP1	EPR3113	Abcam	BX042	1:100
CD16	3G8	ThermoFisher Scientific	BX035	1:50
IBA1	Polyclonal (Novus NB100 1028)	Novus Biologicals	BX037	1:100
CD123	IL3RA/2947R	Abcam	BX050	1:200

Table S3. Staining antibodies for Co-detection by indexing (CODEX).

Spike fw (S-SfaAI_fw)	GCGATCGCCATGTTCGTGTTTCTGGT
S1-rv (S-S1-NotI_rv)	GCGGCCGCTCATCTGGCCCGTCTGG
S1'-rv (S-S1'-NotI_rv)	GCGGCCGCTTACCGCTTGCTGGGC
NRP2SP-HA-rv*	CAGGTTCACACACTGGCTAGCATAATCTGGAACATCATATGGATA
NRP2SP-HA-fw**	TATCCATATGATGTTCCAGATTATGCTAGCCAGTGTGTGAACCTG
In combination with pCMV6 specific vector primers VP1.5 (*) and XL39 (**).	

ACE2 FW2	AGCACTCACGATTGTTGGGA
ACE2 RV2	CCACCCCAACTATCTCTCGC
FUR FW1	TGTGGTGTAGGTGTGGCCTA
FUR RV1	GCTGGCACTGTAGATGTGGA
NRP1 FW	CCCCAAACCACTGATAACTCG
NRP1 RV	GTCCAGACACCATACCCAACAT
NRP2tot FW	ACCAGAACTGCGAGTGGATT
NRP2tot RV	CGATGTTCCCACAGTGTTTG
PPIA FW	GCTGGACCCAACACAAATGG
PPIA RV	GGCCTTCCACAATATTCATGCCT
TMPRSS2 FW1	AGCAAGTGCTCCAACTCTGG
TMPRSS2 RV1	AAGTTTGGTCCGTAGAGGCG

Table S4. Primers used to generate soluble spike fragments and primers used for qRT-PCR (below; see Supplementary Figure S3).

Target	Dilution	Manufacturer	Catalog No.
Primary antibodies			
HA-Tag (C29F4) Rabbit mAb	1:800 in blocking solution	Cell Signaling Technology	3724S
Anti-Neuropilin 1 antibody, rabbit mAb, BSA and Azide free, [EPR3113]	1:500 in blocking solution	Abcam	ab184783
Anti-NRP2 antibody [EPR23808-72], rabbit mAb	1:100 in blocking solution	Abcam	ab273584
Rabbit mAB IgG XP® Isotype Control	1µg/ml in blocking solution	Cell Signaling Technology	3900S
Alexa Fluor^{TM} Plus 405 Phalloidin	0.5µl 400X stock solution in 200µl 1%BSA/PBS	LIFE Technologies Europe BV	A30104
Secondary antibodies			
Cy3-Affini Pure Goat Anti-Rabbit IgG (H+L)	1:250 in blocking solution	Dianova	111-165-144

Table S5. Antibodies, immunofluorescence (see Figure 5, Supplementary Figure S4).

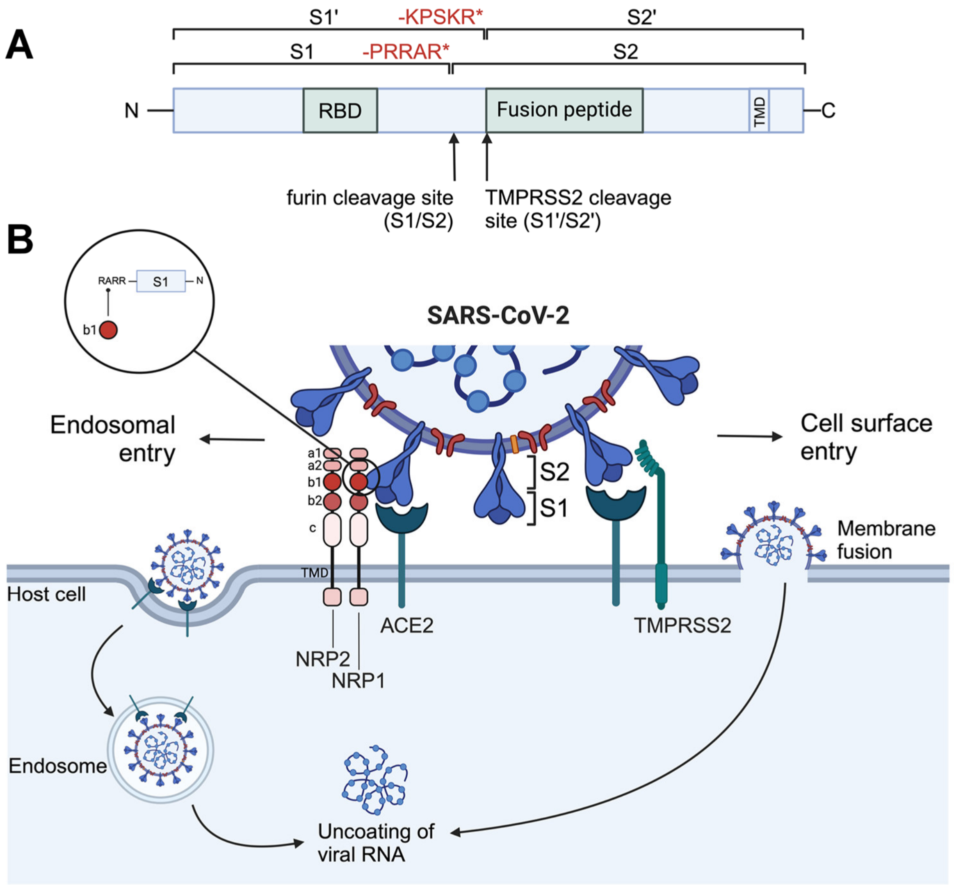

Figure S1. Schematic diagram of SARS-CoV-2 entry into host cells. (A) The S protein contains a new internal proteolytic cleavage site S1/S2, utilized by furin proteases, which generates an N-terminal S1 and a C-terminal S2 fragment. Host cell protease TMPRSS2 cleaves the S protein at the S1‘/2‘ cleavage site, resulting in an N-terminal S1‘ and a C-terminal S2‘ fragment, which exposes the fusion peptide. **(B)** The spike receptor binding domain (RBD) is the primary SARS-CoV-2 attachment site to ACE2 on the host cell. TMPRSS2 is required for subsequent spike protein cleavage to enable viral fusion with the host cell membrane. Cleavage of S protein through host cell furin protease at the S1/S2 cleavage site reveals a C-terminal -RRAR* sequence, to which NRP1 and NRP2 bind via their b1 and b2 domains. TMD: transmembrane domain. Image was created with BioRender.

A

	Median	Mean	Negative, *n/n* (%)	Low, *n/n* (%)	Moderate, *n/n* (%)	High, *n/n* (%)
COVID-19 (n=16)	2	2.38	0/16 (0)	0/16 (0)	10/16 (62.5)	6/16 (37.5)
Influenza (n=7)	2	2.0	0/7 (0)	2/7 (28.57)	3/7 (42.86)	2/7 (28.57)
Control (n=5)	3	2.8	0/5 (0)	0/5 (0)	1/5 (20)	4/5 (80)

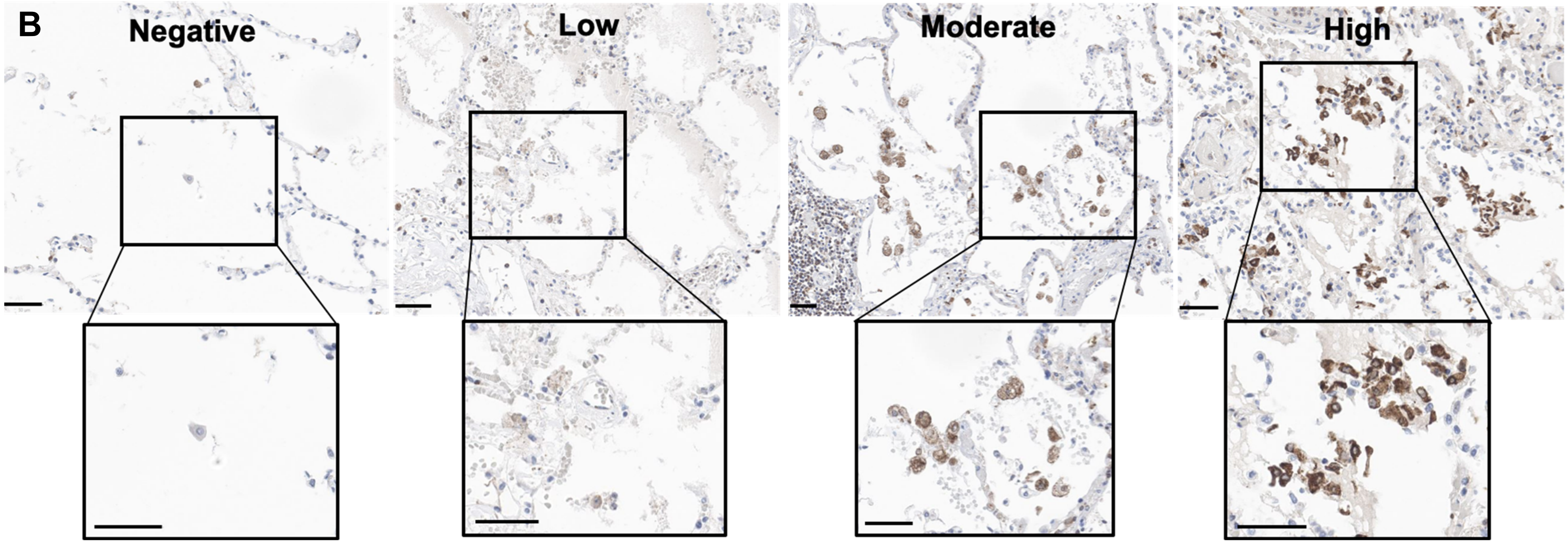

Figure S2. Semiquantitative analysis of NRP1 (IHC), lung. (A) Intensity of NRP1 IHC staining in alveolar macrophages, peripheral lung (see methods). Scoring: 0, no expression/negative; 1, low expression; 2, moderate expression; 3, high expression. Median and mean are shown per group. **(B)** Representative IHC images (NRP1) demonstrating examples of NRP1 expression. Scale bars correspond to 50 μm.

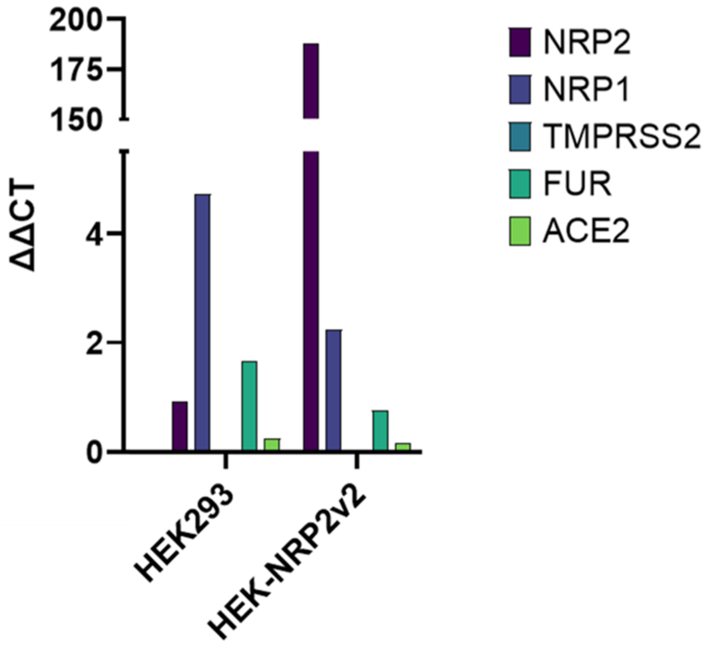

Figure S3. Relative gene expression analysis (ΔΔCτ) in HEK293 cells and NRP2-transfected HEK293 cells. Shown are the mean ΔΔCτ values for the expression of *NRP2*, *NRP1*, *TMPRSS2*, *FURIN (FUR)* and *ACE2* in wild type HEK293 and HEK-NRP2v2 cells (HEK293 cells stably transfected with *NRP2*). Expression levels were normalized to the housekeeping gene *PPIA*, with Caco-2 cells used as the calibrator (not shown). We found negligible expression of known spike interacting proteins like *ACE2*, *FURIN* and *TMPRSS2* in HEK293 cells, while *NRP1* was highly expressed. HEK-NRP2v2 cells overexpressed *NRP2*. qRT-PCR was performed with 10 ng cDNA reverse-transcribed using RevertAid H minus (ThermoFisher Scientific), with Maxima SYBR Green and gene-specific primers (Supplementary Table S4) on a TaqMan 7500 Fast Real-Time PCR System (Applied Biosystems, ThermoFisher Scientific). Reactions were run in triplicate with *PPIA* as the reference, and data were analyzed using the 7500 System Software.

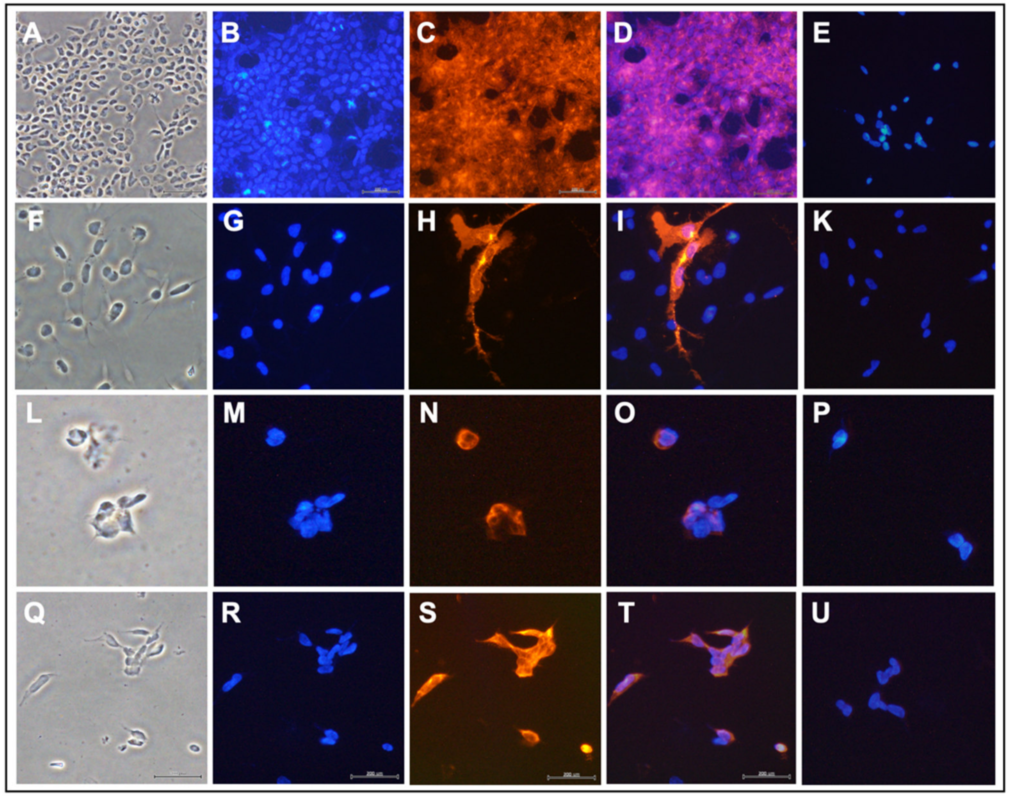

Figure S4. Immunofluorescence (IF) of HEK293 cells stably transfected to express NRP2 or spike fragments S1 or S1'. HEK293 cells endogenously expressing NRP1 **(A-E)**. HEK293 cells stably transfected to express NRP2 **(F-K)**, S1 spike fragment **(L-P)** or S1' spike fragment **(Q-U)**. Brightfield images of HEK293 cells **(A, F, L, Q)**. DAPI nuclear staining **(B, G, M, R)**. IF images of NRP1 **(C)**, NRP2 **(H)** or anti-HA antibody **(N, S)** to detect S1 **(N)** or S1' **(S)**. IF and DAPI stain merges are shown in **D, I, O, T**. Corresponding isotype controls in **E, K, P, U**.

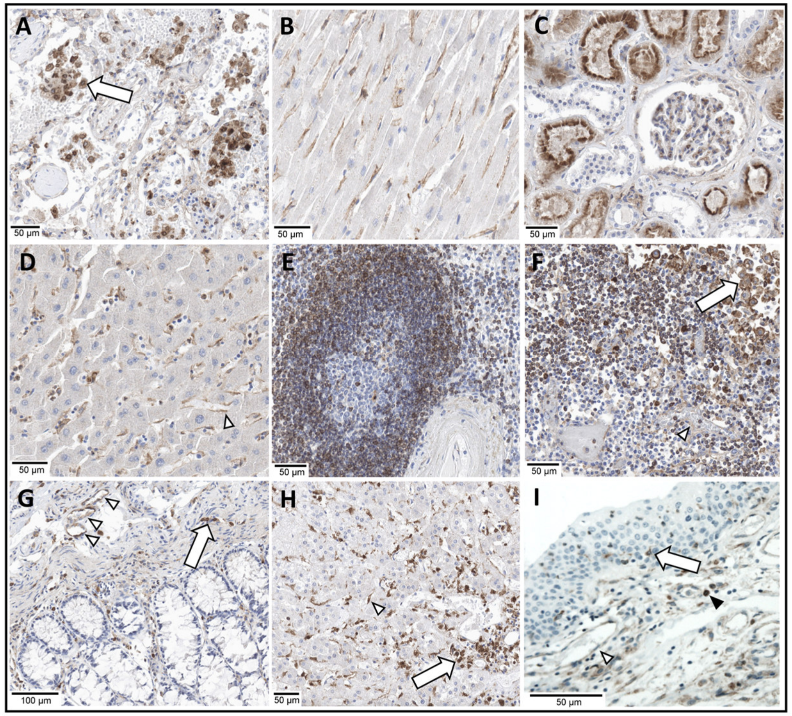

Figure S5. NRP1 expression in fatal COVID-19 (IHC), various organs. Prominent NRP1 expression on alveolar macrophages (arrow) and interstitial macrophages **(A)**, while EC were sparsely and heterogeneously positive. In the heart, capillary EC expressed abundant NRP1 **(B)**. In kidneys, we observed NRP1 expression in proximal tubules as well as renal corpuscles (Bowman's membrane and glomeruli) **(C)**. Kupffer cells and few sinusoidal EC (arrowhead) were positive for NRP1 in the liver **(D)**. In spleen **(E)** and lymph nodes **(F)**, lymphocytes in lymph follicles were strongly positive for NRP1. In lymph node, vascular endothelial vessels were positive for NRP1 (arrowhead). Sinus histiocytes in lymph node strongly expressed NRP1 (arrow) **(F)**. NRP1 was also present in vessels in the submucosa (arrowhead) of the large bowel as well as in lamina propria mononuclear cells **(G)**. In the adrenal gland, endothelium (arrowhead) and macrophages (arrow) highly expressed NRP1 **(H)**. The trachea **(I)** showed NRP1 expression in vessels of submucosa (white arrowhead) and mononuclear cells (black arrowhead). Metaplastic respiratory epithelium showed few superficial and basal cells expressing NRP1 (not in image). In the epithelium, morphologically identified dendritic cells with processes were positive for NRP1 (arrow). Regions were photographed using QuPath and are taken from patient 1 (F, H), 2 (A–E), 3 (G) and 6 (I), see Supplementary Table S1A.

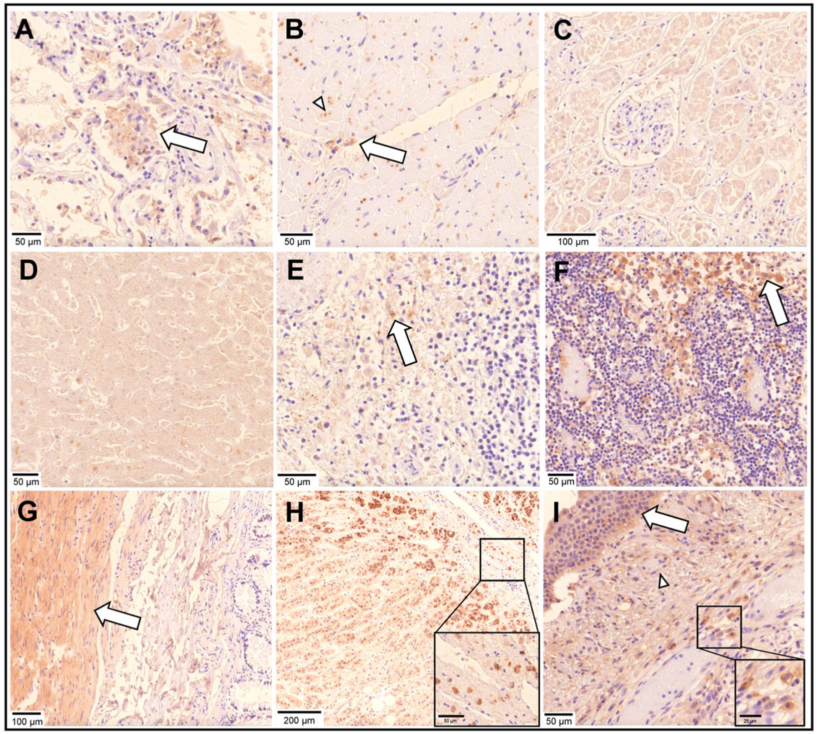

Figure S6. NRP2 expression in fatal COVID-19 (IHC), various organs. In the lung, alveolar macrophages (arrow) and interstitial macrophages were weakly positive for NRP2 **(A)**, EC were rarely positive for NRP2. In the heart, capillaries and endothelium of larger vessels was negative for NRP2, intracytoplasmatic staining reflects lipofuszine in cardiomyocytes (**B**, arrowheads). NRP2 was expressed in scattered sparse mononuclear cells (arrow). In kidneys, we observed NRP2 on few cells in glomeruli, while tubules were negative **(C)**. Hepatocytes were negative for NRP2 **(D)**. In the spleen **(E)**, sparse scattered mononuclear cells in the red pulp and pigment-laden macrophages (arrow) were positive for NRP2. In lymph nodes **(F)**, sinus histiocytes were strongly positive for NRP2 (arrow). Also, lymphatic endothelium of cortical sinuses weakly stained for NRP2 (not in image). NRP2 was strongly present in smooth muscle cells (arrow) and in mononuclear cells in lamina propria of the colon **(G)**. In the adrenal gland **(H)**, cortical epithelium especially in the zona reticularis highly expressed NRP2. Macrophages (shown in inset) were also positive for NRP2. The trachea **(I)** showed weak NRP2 expression in vessels of submucosa (arrowhead) and mononuclear cells (inset) as well as in the respiratory epithelium (arrow). Regions were photographed using QuPath and are taken from patient 1 (E–H), 2 (I) and 3 (A–D), see Supplementary Table S1A.

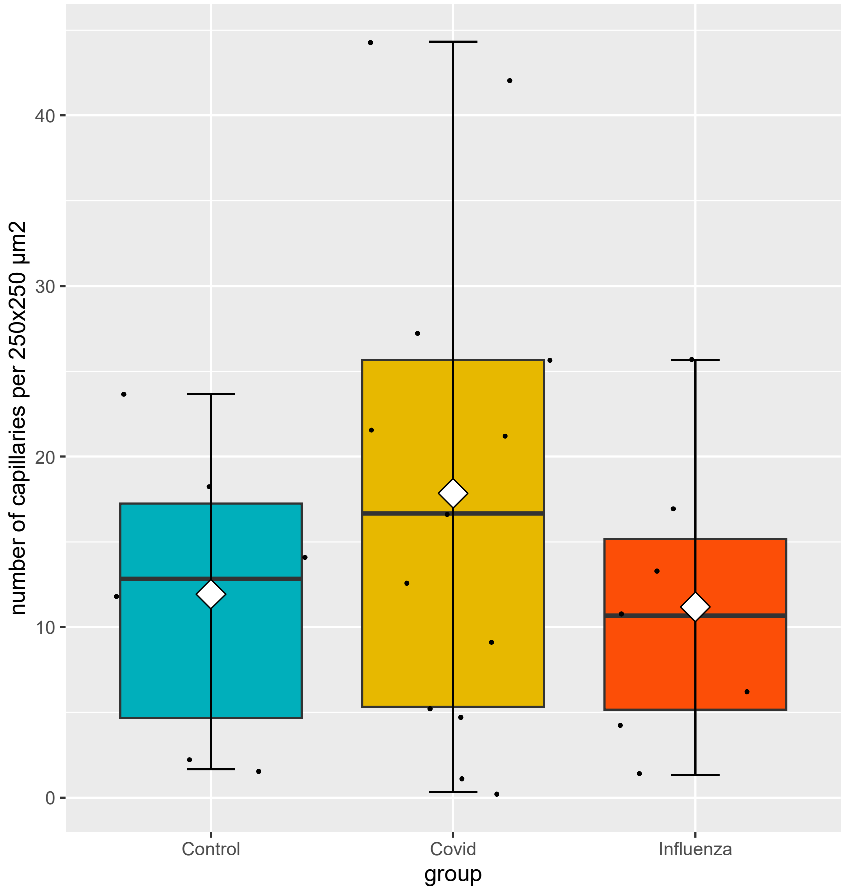

Figure S7. Quantitative analysis of NRP1 (IHC), heart. Manual counting of NRP1-positive capillaries per 0,0625 mm^2 square in COVID-19 (X = 17.85, SD = 14.40, n = 13), influenza pneumonitis (X = 11.19, SD = 8.37, n = 7) and controls (X = 11.95, SD = 8.72, n = 6). Median is depicted as a black line, mean (x) is depicted as a white rhombus. ANOVA yielded no significant difference between groups ($F(2, 23) = 0.91$, $p = 0.417$).

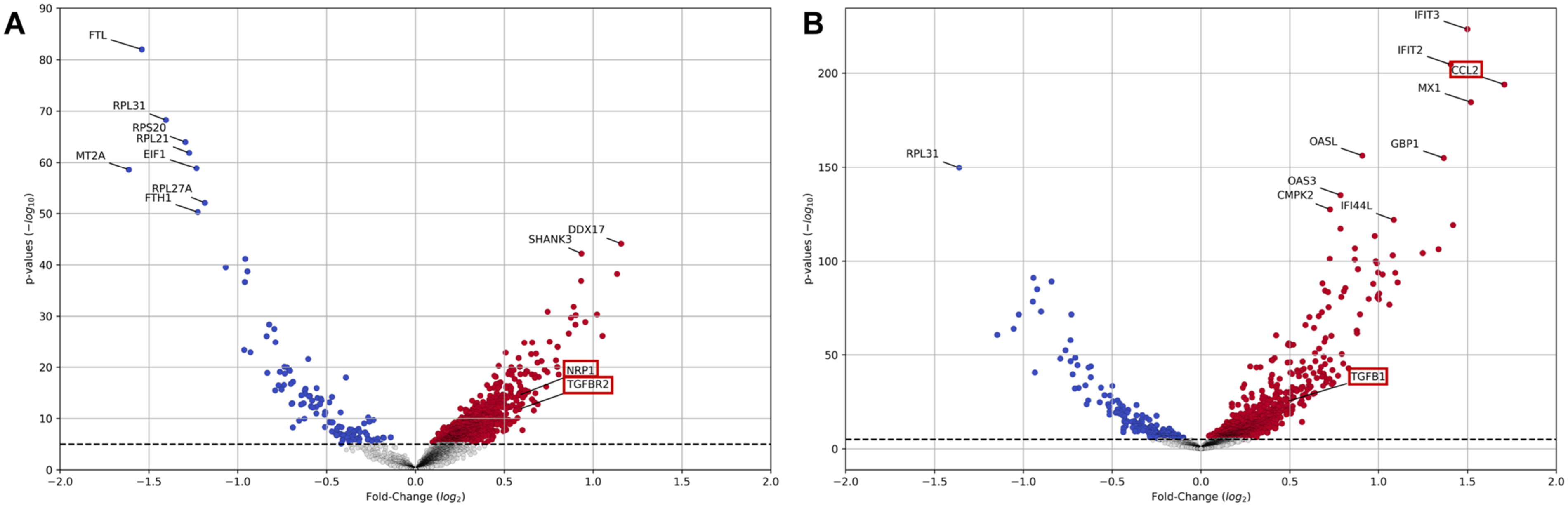

Figure S8. Differential gene expression in fatal COVID-19 versus non-infectious controls, lung. (A) Subcluster of vascular EC. **(B)** Subcluster of monocytes. Results are from a single cell-based differential expression model by Delorey et al. (2021) (1). The ten genes with the highest negative log10 *p* values are annotated. Additionally, *NRP1*, *TGFBR2, CCL2* and *TGFB1* are boxed. A positive log2 fold change indicates upregulation of the gene (red), a negative log2 fold change indicates downregulation (blue). Horizontal dashed line, $p < 1E\text{-}5$. *FTL*: ferritin light chain, *RPL31*: ribosomal protein L31, *RPS20*: ribosomal protein S20, *RPL21*: ribosomal protein L21, *EIF1*: eukaryotic translation initiation factor 1, *MT2A*: metallothionein 2A, *RPL27A*: ribosomal protein L27a, *FTH1*: ferritin heavy chain 1, *SHANK3*: SH3 and multiple ankyrin repeat domains 3, *DDX17*: DEAD-box helicase 17, *NRP1*: neuropilin 1, *TGFBR2*: transforming growth factor beta receptor 2, *IFIT3*: interferon induced protein with tetratricopeptide repeats 3, *IFIT2*: interferon induced protein with tetratricopeptide repeats 2, *CCL2*: C-C motif chemokine ligand 2, *MX1*: MX dynamin like GTPase 1, *GBP1*: guanylate binding protein 1, *OASL*: 2'-5'-oligoadenylate synthetase like, *OAS3*: 2'-5'-oligoadenylate synthetase 3, *CMPK2*: cytidine/uridine monophosphate kinase 2, *IFI44L*: interferon induced protein 44 like, *TGFB1*: transforming growth factor beta 1.

References

1. Delorey TM, Ziegler CGK, Heimberg G, Normand R, Yang Y, Segerstolpe Å, Abbondanza D, Fleming SJ, Subramanian A, Montoro DT, Jagadeesh KA, Dey KK, Sen P, Slyper M, Pita-Juárez YH, Phillips D, Biermann J, Bloom-Ackermann Z, Barkas N, Ganna A, Gomez J, Melms JC, Katsyv I, Normandin E, Naderi P, Popov YV, Raju SS, Niezen S, Tsai LT-Y, Siddle KJ, Sud M, Tran VM, Vellarikkal SK, Wang Y, Amir-Zilberstein L, Atri DS, Beechem J, Brook OR, Chen J, Divakar P, Dorceus P, Engreitz JM, Essene A, Fitzgerald DM, Fropf R, Gazal S, Gould J, Grzyb J, Harvey T, Hecht J, Hether T, Jané-Valbuena J, Leney-Greene M, Ma H, McCabe C, McLoughlin DE, Miller EM, Muus C, Niemi M, Padera R, Pan L, Pant D, Pe'er C, Pfiffner-Borges J, Pinto CJ, Plaisted J, Reeves J, Ross M, Rudy M, Rueckert EH, Siciliano M, Sturm A, Todres E, Waghray A, Warren S, Zhang S, Zollinger DR, Cosimi L, Gupta RM, Hacohen N, Hibshoosh H, Hide W, Price AL, Rajagopal J, Tata PR, Riedel S, Szabo G, Tickle TL, Ellinor PT, Hung D, Sabeti PC, Novak R, Rogers R, Ingber DE, Jiang ZG, Juric D, Babadi M, Farhi SL, Izar B, Stone JR, Vlachos IS, Solomon IH, Ashenberg O, Porter CBM, Li B, Shalek AK, Villani A-C, Rozenblatt-Rosen O, Regev A. 2021. COVID-19 tissue atlases reveal SARS-CoV-2 pathology and cellular targets. Nature 595:107–113.
